# Sediment fluxes rather than oxic methanogenesis explain diffusive CH_4_ emissions from lakes and reservoirs

**DOI:** 10.1038/s41598-018-36530-w

**Published:** 2019-01-18

**Authors:** Frank Peeters, Jorge Encinas Fernandez, Hilmar Hofmann

**Affiliations:** Limnological Institute, University of Konstanz, Mainaustrasse 252, D-78464 Konstanz, Germany

## Abstract

Methane emissions from lakes and reservoirs are a major natural source in the global budget of atmospheric CH_4_. A large fraction of these emissions are due to diffusive transport of CH_4_ from surface waters to the atmosphere. It was suggested recently that CH_4_ production in the oxic surface waters is required to compensate for diffusive CH_4_ emissions from lakes. In contrast, we demonstrate here that typical diffusive CH_4_-fluxes from sediments in shallow water zones, *F*_*sed,S*_, suffice to explain CH_4_ emissions to the atmosphere. Our analysis is based on the combination of an exceptional data set on surface concentrations of CH_4_ with a mass balance model of CH_4_ that is focused on the surface mixed layer and considers CH_4_-fluxes from sediments, lateral transport, gas exchange with the atmosphere, and includes temperature dependencies of sediment fluxes and gas exchange. *F*_*sed,S*_ not only explains observed surface CH_4_ concentrations but also concentration differences between shallow and open water zones, and the seasonal variability of emissions and lateral concentration distributions. Hence, our results support the hypothesis that diffusive fluxes from shallow sediments and not oxic methanogenesis are the main source of the CH_4_ in the surface waters and the CH_4_ emitted from lakes and reservoirs.

## Introduction

Methane (CH_4_) is a very potent greenhouse gas and emissions from lakes and reservoirs constitute a major natural source in the global budget of atmospheric CH_4_^1,2^. Several sources and transport pathways of CH_4_ in lake waters have been identified^3–11^. Typically, CH_4_ is produced within anoxic sediments^12^ and oxidized at anoxic-oxic interfaces by CH_4_ oxidizing bacteria^13–16^. Anaerobic production of CH_4_ in sediments and CH_4_ emissions from ecosystems increase with increasing water temperature at a similar rate and the temperature dependence can be described by the Boltzmann-Arrhenius law with an apparent activation energy of *E*_*a*_ = 0.82–1.07 eV^17^. CH_4_ emission from lakes increase exponentially with temperature at an exponent of 0.13 °C^−1 ^^18^, approximately corresponding to an activation energy of 0.90 eV.

The main pathways of CH_4_ emissions from lakes are ebullition of CH_4_-rich gas bubbles released from oversaturated sediments and diffusive exchange of CH_4_ between water and atmosphere at the lake surface. Diffusive exchange typically accounts for about 40% to 50% of the total CH_4_ emissions from lakes to the atmosphere^4,19^ and is the focus in the study here. Diffusive gas transport to the atmosphere is proportional to the atmospheric equilibrium concentration, the concentration of the gas in the surface water, and the gas transfer velocity (e.g.^20^).

CH_4_ concentrations in the surface water and CH_4_ emissions change seasonally^21,22^ as they increase with water temperature^17,18,23^ and are affected by seasonal mixing^24–26^. Furthermore, surface water concentrations of CH_4_ are spatially not homogeneous^19,22^ but typically enriched in shallow water zones^3,23,27,28^. Thus, diffusive CH_4_ emissions from lakes vary in space and time.

The origin of the CH_4_ in the surface water of lakes is currently under debate. Several studies have suggested production of CH_4_ in oxic surface waters^7,29^. Recently it has been claimed that oxic CH_4_ production is a major source of CH_4_ in the surface waters of lakes^9^ and that production of CH_4_ in oxic surface waters is required to compensate the loss of CH_4_ due to diffusive emissions^30^. Other studies have explained the comparatively high concentrations of CH_4_ in surface waters of lakes by lateral transport of CH_4_-rich waters from shallow water zones^23,27,28^. In shallow water zones CH_4_ concentrations can be enriched due to mobilization of pore water during resuspension events^27^, the presences of plants^3,31,32^, and/or because of the temperature dependence of CH_4_ production in sediments and the comparatively high temperatures of littoral sediments^33,34^. Statistical analysis of a large data set on surface CH_4_ distributions in several lakes revealed that basin wide average CH_4_ concentrations are described better by the ratio of the area of the shallow water zone to the area of the entire surface than by surface area alone^23^. Based on these observations Encinas *et al*.^23^ suggested that diffusive fluxes from sediments in the shallow water zone are a major source of surface water CH_4_ and diffusive CH_4_ emissions from lakes. However, this hypothesis was not tested by a quantitative analysis comparing sediment fluxes and emissions.

Donis *et al*.^30^ employed a full mass balance of CH_4_ in Lake Hallwil to quantitatively compare the losses of CH_4_ by diffusive emissions at the lake surface with diffusive CH_4_ fluxes from sediments, CH_4_ oxidation and other sources and sinks of CH_4_. Donis *et al*.^30^ claim, that the amount of CH_4_ emitted at the lake surface exceeds the CH_4_ provided by diffusive fluxes from sediments by a factor of 26. They conclude that a large additional source of CH_4_ is required and hypothesize that the major part of this source is production of CH_4_ in the open water providing 22 times more CH_4_ than the total diffusive fluxes from the sediments in the surface mixed layer. This argument implies that without the additional source of CH_4_, i.e. without net-production in the mixed surface layer, diffusive fluxes from the sediments in the surface mixed layer, *F*_*sed,S*_, required to compensate the total diffusive emissions of CH_4_ from the lake surface would be extremely large (>40 mmol m^−2^ d^−1^; using the sediment flux of Donis *et al*.^30^) and beyond reasonable values expected from measured sediment fluxes. We test this conclusion by estimating *F*_*sed,S*_ required to provide sufficient CH_4_ to compensate total diffusive CH_4_ emissions to the atmosphere, *E*_*atm*_, in several lake basins and reservoirs. We compare these values of required *F*_*sed,S*_ with the typical range of measured *F*_*sed,S*_ in other system and with the diffusive sediment flux *F*_*sed,Hal*_ obtained from the analysis of pore water concentrations measured by Donis *et al*.^30^ in a sediment core collected in the surface mixed layer of Lake Hallwil.

In our analysis we combine a mass balance model for CH_4_ in the mixed surface layer with one of the largest data sets on CH_4_ distributions within lakes and reservoirs. The CH_4_ mass balance model considers as source of CH_4_ diffusive fluxes from the sediments, loss of CH_4_ due to diffusive emissions from the water surface to the atmosphere, temperature dependence of these sources and losses, and lateral transport of CH_4_ by turbulent mixing within the surface mixed layer. We demonstrate that, in contrast to the conclusion from the argument by Donis *et al*.^30^, *F*_*sed,S*_ required to explain *E*_*atm*_ is not ~20 times larger but on average smaller than *F*_*sed,Hal*_. This result suggests that net-production of CH_4_ in the surface mixed layer is not required to close the mass balance of CH_4_. However, this argument has the weakness that it compares atmospheric emissions with sediment fluxes from a different system. We therefore additionally re-analyzed the data of Donis *et al*.^30^ and confirm that also in Lake Hallwil the measured diffusive sediment flux *F*_*sed,Hal*_ provides sufficient CH_4_ to compensate the total diffusive losses of CH_4_ to the atmosphere from this system. Finally, we dynamically simulate the CH_4_ development along a transect in Lake Uberlingen over several seasons. We demonstrate that the simple model, which does not include net-production of CH_4_ in the water, is sufficient to adequately describe the seasonal development of CH_4_ concentrations and the seasonal changes in the lateral distribution of CH_4_.

## Results

**Figure 1:**
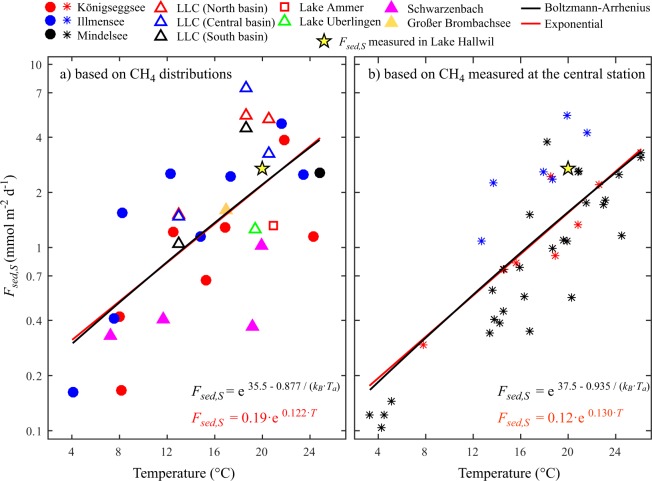
Diffusive CH_4_ fluxes from the sediments of the shallow water zone, *F*_*sed,S*_, required to compensate total diffusive CH_4_ emissions to the atmosphere. *F*_*sed,S*_ were calculated based on spatially averaged CH_4_ concentrations in the surface water utilizing spatially highly resolved distributions of CH_4_ available from numerous campaigns on several lakes and reservoirs (**a**), and on time series of CH_4_ concentrations measured in the surface water at the center of several lakes (**b**). The diffusive sediment flux derived from pore-water measurements in Lake Hallwil, *F*_*sed,Hal*_, is shown by a yellow star. The temperature dependence of *F*_*sed,S*_ can be well described by the Boltzmann-Arrhenius law (black regression lines) or an exponential law (red regression lines). The degree of freedom *df* is 29 and 48 in (**a**) and (**b**), respectively.

The diffusive fluxes of CH_4_ from sediments in shallow waters, *F*_*sed,S*,_ estimated for all campaigns in all lakes and reservoirs for which spatially well resolved CH_4_ data were available, range between 0.16 and 7.4 mmol m^−2^ d^−1^ (Fig. 1a) with an average *F*_*sed,S*_ of 2.0 mmol m^−2^ d^−1^ and a standard deviation of 1.8 mmol m^−2^ d^−1^. These sediment fluxes were determined assuming steady state conditions and are the sediment fluxes sufficient to compensate the total diffusive emissions of CH_4_ from the respective lakes and reservoirs to the atmosphere.

*F*_*sed,S*_ increases strongly with increasing water temperature (Fig. 1). The temperature dependence can be well described by Boltzmann–Arrhenius law with an apparent activation energy *E*_*a*_ = 0.877 eV (*R*^2^ = 0.47, *df* = 29, *p* < 0.001). This activation energy agrees well with that determined for CH_4_ production in sediments and CH_4_ ecosystem emissions^17^. Note that the temperature dependence of *F*_*sed,S*_ can be described similarly well using an exponential function with an exponent of 0.122 °C^−1^ (*R*^2^ = 0.46, *df* = 29, *p* < 0.001) which closely agrees with the temperature dependence of CH_4_ emissions from surface waters of lakes^18^.

The overall range of the values for *F*_*sed,S*_ is about the same as the range of published CH_4_ diffusive fluxes from surface sediments (0.03 to ~7 mmol m^−2^ d^−1^) that were estimated based on measured CH_4_ concentration gradients in the pore water of sediment cores^33,35^ or on measurements of the CH_4_ flux from sediment cores into overlying water^36^. However, the wide range of values is partially due to the temperature dependence of *F*_*sed,S*_.

At 20 °C the relation of *F*_*sed,S*_ as function of temperature provides on average *F*_*sed,S*_(20 °C) = 2.2 mmol m^−2^ d^−1^ (Fig. 1a). This value is ~20% smaller than the diffusive sediment flux *F*_*sed,Hal*_ = 2.8 mmol m^−2^ d^−1^ determined from the re-analysis (see Supplementary Section S3) of the pore water concentrations measured by Donis *et al*.^30^ in the sediment core collected at 3 m water depth in Lake Hallwil (yellow star in Fig. 1a,b). Thus, in the lakes considered in our study the sediment fluxes required to compensate total diffusive emissions to the atmosphere at 20 °C are smaller than the diffusive sediment flux measured in Lake Hallwil, i.e. on average the required sediment fluxes are smaller than *F*_*sed,Hal*_ = 2.8 mmol m^−2^ d^−1^. The largest sediment fluxes required to compensate total diffusive emissions are at most 2.5 times larger than *F*_*sed,Hal*_ (i.e. in LLC). These results are in conflict with the CH_4_ mass balance of Donis *et al*.^30^ for Lake Hallwil. According to Donis *et al*.^30^ the total diffusive CH_4_ emission to the atmosphere is ~26 time larger than the total CH_4_ flux from the littoral sediments (Table 2 in Donis *et al*.^30^: 5040 mol d^−1^ versus 196 mol d^−1^) which implies that in Lake Hallwil *F*_*sed,S*_ required to compensate total CH_4_ emissions to the atmosphere is ~26 times larger than the sediment flux Donis *et al*.^30^ obtained from their pore water measurements. However, our re-analysis of the CH_4_ mass balance in the surface mixed layer of Lake Hallwil reveals that the *F*_*sed,S*_ required to compensate the total diffusive CH_4_ emissions to the atmosphere in Lake Hallwil is only *F*_*sed,S*_ = 2.5 to 2.7 mmol m^−2^ d^−1^, which is smaller and not orders of magnitude larger than the measured CH_4_ flux from the sediments *F*_*sed,Hal*_ = 2.8 mmol m^−2^ d^−1^ (a detailed re-evaluation of the data from Lake Hallwil is provided in the Supplementary Section S3). Thus the CH_4_ mass balance in Lake Hallwil implies that *F*_*sed,Hal*_ is sufficient to compensate the total diffusive emissions at the surface of Lake Hallwil. The total source of CH_4_ in the surface mixed layer due fluxes from the sediments *S*_*sed,total*_ = 1990 mol d^−1^ is slightly larger than the total loss of CH_4_ due to emissions to the atmosphere at 20 °C (between 1800 and 1900 mol d^−1^, see Supplementary Section S3). Apparently, the mass balance in Lake Hallwil does not require an unknown process producing substantial amounts of CH_4_ in the open water (for details see Supplementary Section S3).

*F*_*sed,S*_ required to compensate total diffusive CH_4_ emissions at the lake surface were also estimated using surface water CH_4_ concentrations measured at the center of several lakes (Fig. 1b) assuming that these CH_4_ concentrations are representative for the lake-wide average surface concentration. The temperature dependence of *F*_*sed,S*_ derived from the CH_4_ concentrations in the center of the lakes (Fig. 1b) and from the average surface concentration (Fig. 1a) is essentially the same. However, the *F*_*sed,S*_ in Fig. 1b are generally smaller than those in Fig. 1a. This difference may be explained by the fact that the surface concentration at the center of a lake is typically smaller than the lake-wide average surface concentration of CH_4_^23^. However, the combination of data from different lakes and reservoirs may also contribute to the difference between the values of *F*_*sed,S*_ in Fig. 1a,b

*F*_*sedS*_ is the flux per unit time and unit sediment area from sediments in the shallow water zone which provides sufficient CH_4_ that the overall flux from the sediments in the shallow water zone (*F*_*sedS*_ · A_s_) compensates the overall flux from the lake surface into the atmosphere ($\bar{{F}_{{atm}}}$·*A*_*Surf*_). The calculation of *F*_*sedS*_ assumes that the CH_4_ in the surface mixed layer originates only from sediments of the shallow water zone and has no source in the open water. In this case CH_4_ concentrations should be larger in the shallow than in the open water zone. If, however, all methane is produced by oxic methanogenesis within the water column of the surface mixed layer one would expect the opposite, i.e. larger CH_4_ concentrations in the open water than in the shallow water zones, because CH_4_ production per unit surface area is larger in the open water than in the near shore zones where the water depth is smaller than the depth of the surface mixed layer.

**Figure 2:**
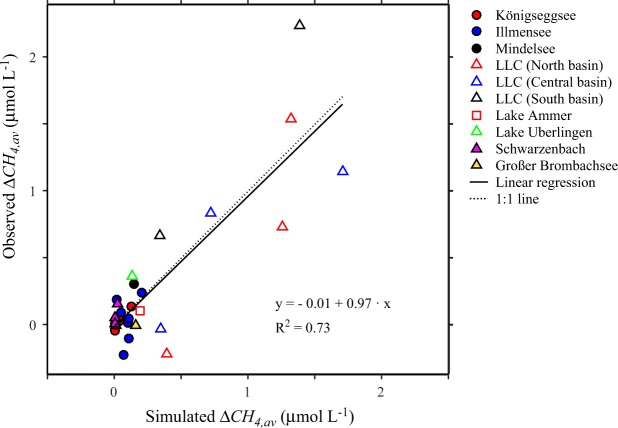
Comparison of observed and simulated ∆*CH*_*4,av*_. ∆*CH*_*4,av*_ is the difference between the average concentration in the shallow and the average concentration in the open water. The model results are obtained from simulations assuming steady state conditions. The regression line has an intercept of (−0.01 ± 0.06) mmol L^−1^ and a slope of 0.97 ± 0.10 and explains 73% of the variance (*R*^2^ = 0.73, *df* = 29). The intercept does not differ from zero (*p* = 0.9) whereas the slope significantly differs from zero but not from one (*p* < 0.001, *p*_1_ = 0.8).

To test whether the estimated sediment fluxes *F*_*sed,S*_ explain the observed horizontal distribution of CH_4_ within the surface mixed layer of the basins, the model was applied to simulate the concentration distribution of CH_4_ at steady state for each campaign assuming radial symmetry of the basins and by using the *F*_*sed,S*_ of the respective basin and time depicted in Fig. 1a. The difference between the average CH_4_ concentration in the shallow and the average CH_4_ concentration in the open water, ∆*CH*_*4,av*_, was determined from the model results and from the observations. Observed and simulated ∆*CH*_*4,av*_ agree well and both indicate that CH_4_ concentrations in the surface mixed layer are typically larger in shallow near shore than in the open water zones (Fig. 2). The regression line has a slope of 0.97 and differs significantly from zero but not from 1 (*p* < 0.001, *p*_1_ = 0.8), and the intercept is −0.01 μmol L^−1^ and does not significantly differ from 0 (*p* = 0.9). The results from the regression analysis support the conclusion that the concentration differences between shallow and open water can be explained by the assumption that the source of CH_4_ in the surface waters of lakes and reservoirs is the CH_4_ flux from sediments in shallow waters.

**Figure 3:**
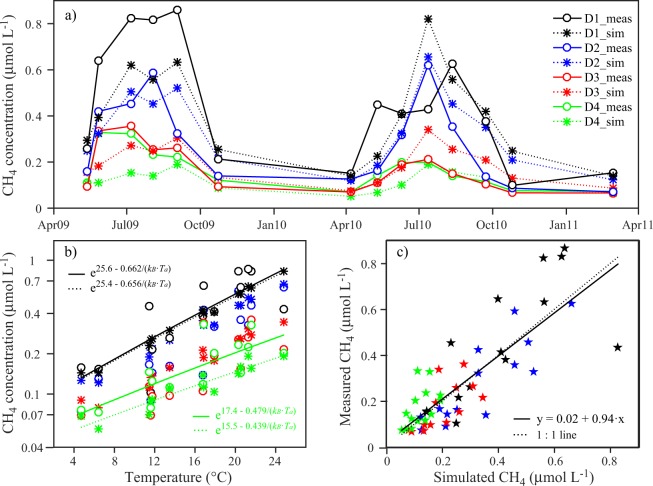
Comparison of observations with results from a long-term simulation of CH_4_ concentrations in the surface mixed layer along a transect in Lake Uberlingen. Time series of simulated (sim) and measured (meas) CH_4_ concentrations are depicted in (**a)** distinguishing between four distance ranges from shore (different colors). Distance ranges from shore are defined as: D1: <100 m, D2: 100–300 m, D3: 300–1000 m, D4: 1000–1850 m distance from shore. (**b**) Simulated and measured concentrations increase with water temperature at similar rates. (**c**) Model results and observations agree well and consistently indicate that concentrations are typically larger in near shore regions and decrease towards the open water with distance from shore. The degree of freedom *df* is 53 and 54 in (**a**) and (**c**), respectively.

The statistical analysis of measured and simulated differences between shallow and open water CH_4_ concentrations combining several lakes and reservoirs could be affected by differences between these systems in trophic state or other properties influencing CH_4_.We therefore have included an analysis which focuses on data from a single large lake, Lake Uberlingen, and applied the model to dynamically simulate the temporal development of the CH_4_ distribution over two years. Measured and simulated seasonal changes in the CH_4_ concentrations agree well (Fig. 3a). Furthermore, nearshore CH_4_ concentrations are typically larger than the concentrations at larger distance from shore, and simulated and measured concentrations at the same distance from shore agree well with each other (Fig. 3a). Note that the simulation results are derived from a time continuous model that required only three time constant parameters. Inverse fitting of the model to the 56 data points provides as best fit parameters the dispersion coefficient *K*_*h,disp*_ = 1.4 m^2^ s^−1^, and *E*_*a*_* = *0.823 eV and *C* = 33.6 of the Boltzmann-Arrhenius law. The diffusive CH_4_ flux from the shallow water sediments obtained from these parameters increases with temperature and is *F*_*sed,S*_ = 2.8 mmol m^−2^ d^−1^ at 20 °C. This value of *F*_sed,S_, which is the sediment flux sufficient to compensate CH_4_ emissions from Lake Uberlingen to the atmosphere, is the same as the sediment flux *F*_*sed,Hal*_ = 2.8 mmol m^−2^ d^−1^ obtained from the pore-water measurements^30^ in Lake Hallwil. The dispersion coefficient obtained by the inverse fitting, *K*_*h,disp*_ = 1.4 m^2^ s^−1^, is slightly larger than the dispersion coefficient provided by the empirical equation of Lawrence *et al*.^37^ for a length scale of half the transect length (*K*_*h,disp*_ = 1.3 m^2^ s^−1^ at *L* = 1850 m).

CH_4_ concentrations in the surface water increase with water temperatures (*E*_*a*_ ~ 0.66 eV in the shallow water zone and *E*_*a*_ ~ 0.48 eV in the open deep water, Fig. 3b), but at a smaller rate than the sediment fluxes. Figure 3c directly compares data and model results at different distances from shore illustrating that the highest simulated and measured concentrations occur closest to shore and agree well with each other. According to linear regression of measured versus simulated concentrations the model explains 66% of the variance (*R*^2^ = 0.66, *df* = 54) and the regression line has a slope of 0.94 ± 0.09 that does not differ significantly from 1 (*p*_1_ = 0.50) and an intercept of 0.02 ± 0.03 that does not differ significantly from 0 (*p = *0.47).

## Discussion

In spite of the simplifications in the model, the simulated CH_4_ concentrations agree well with field data. Because we assume that net-production of CH_4_ is zero the *F*_*sed,S*_ determined from the model are the diffusive sediment fluxes that are sufficient to compensate diffusive emissions to the atmosphere. *F*_*sed,S*_ are within the range of measured diffusive sediment fluxes^33,35,36^ and are at 20 °C on average smaller than the sediment flux obtained from the pore-water measurements by Donis *et al*.^30^ in Lake Hallwil. This implies that in the systems investigated by us, diffusive sediment fluxes on the same order as the sediment flux in Lake Hallwil, *F*_*sed,Hal*_ = 2.8 mmol m^−2^ d^−1^, result in a total flux of CH_4_ from the sediments in the surface mixed layer that is sufficient to compensate the total diffusive flux of CH_4_ from the lake surface to the atmosphere at 20 °C. Hence, these sediment fluxes suffice to close the mass balance of CH_4_ without requirement of substantial net-production. This conclusion is in conflict with the central argument of Donis *et al*.^30^ who claimed that the total diffusive emissions from the surface of Lake Hallwil are 26 times larger than the total source of CH_4_ due to the flux from sediments in the surface mixed layer and that therefore substantial net-production in open water is required to close the mass balance.

The temperature dependence of *F*_*sed,S*_ obtained from our analysis agrees well with the temperature dependence of CH_4_ production in sediments and emissions from ecosystems and lakes^17,18^. *F*_*sed,S*_ increases with temperature at a larger specific rate than the CH_4_ concentration in the surface water (Fig. 3b). Because the gas transfer velocity increases with temperature, the CH_4_ emissions to the atmosphere must increase with temperature at a larger specific rate than the CH_4_ concentrations in the surface water. Closure of the CH_4_ mass balance requires that the temperature dependence of *F*_*sed,S*_ and of the emissions are similar and thus both must have a larger specific rate of increase with *T* than that of CH_4_ concentrations, which is consistent with the results of our model.

At same temperature *F*_*sed,S*_ differs between lakes and reservoirs which may be explained by different trophic states, properties of the sediments, e.g. porosity or grain sizes, exposure of the sediments to currents or biotic factors such as biofilms, macrophytes or reed belts. For example at ~20 °C *F*_*sed,S*_ in Schwarzenbach reservoir is smaller than in all other lakes and reservoirs investigated which may be explained by the comparatively low alkalinity and low pH of the water in this reservoir and by the comparatively thin sediment layer that was dry in 1997 when the reservoir was emptied. These factors may have negative effects on CH_4_ production. The sediment fluxes *F*_*sed,S*_ are largest in the three basins of LLC possibly because these basins have particularly large reed belts in the shallow water zone^38,39^.

Modelled and measured ∆*CH*_4*,av*_ agree well and indicate that the CH_4_ concentrations in the shallow near shore zone are typically larger than the CH_4_ concentrations in the open water (Fig. 2). This supports the hypothesis that the main source of the CH_4_ in the surface mixed layer are the sediments in the shallow water zone and not production within the water column. The statistical analysis of measured versus simulated ∆*CH*_*4,av*_ is dominated by the results for the basins of LLC because ∆*CH*_*4,av*_ are large due to the high sediment fluxes, the comparatively large surface area and the large ratio of shallow to open water surface area in LLC. In the smaller lakes, e.g., Illmensee and Königseggsee, ∆*CH*_*4,av*_ is small because at small spatial scales concentration differences are more rapidly homogenized by horizontal mixing than at large spatial scales (see Supplementary Section S2).

The potential influence of differences in the conditions in the lakes and reservoirs on the conclusion of *F*_*sed,S*_ and its temperature dependence has been circumvented in the investigation of the seasonal development of CH_4_ along a transect in Lake Uberlingen. Although only three time-constant parameters of the simplified model were fitted, the simulated CH_4_ concentrations show a very similar temporal development as the field data over the observation period of more than two years. Furthermore, the results obtained from the transect in Lake Uberlingen agree well with the results from all other lakes and reservoirs studied. In all systems reasonable fluxes from sediments in shallow waters suffice to explain surface concentrations and emissions of CH_4_. Hence, our findings suggest that CH_4_ production in oxic surface waters is not required to compensate emissions and may be not the main source of CH_4_ in surface waters of lakes and reservoirs as was claimed recently^9,30^.

Support for CH_4_ production under oxic conditions comes from mesocosm experiments in which CH_4_ concentrations remained essentially constant over 28 days^9^. Rates of oxic CH_4_ production were estimated by assuming that emissions from the mesocosms and oxidation of CH_4_ were compensated by net-production^9^. However, these experiments do not prove that oxic CH_4_ production is a large source of CH_4_ in the unbounded open water of lakes. CH_4_ concentrations in the mesoscosms remained ~ 4–10 times smaller than in the lake water outside the mesocosms suggesting that the mesocosms excluded the major source of CH_4_, e.g. CH_4_ fluxes from littoral sediments. Assuming that CH_4_ is produced in oxic waters by acetoclastic production Bogard *et al*.^9^ have taken a correlation between Chl-*a* and CH_4_ as evidence for the importance of methanogenesis in oxic surface waters in lakes. However, the data set of Bogard *et al*.^9^ only provides a significant correlation between Chl-*a* and CH_4_ if marine systems and freshwater lakes are combined but not for freshwater lakes alone^23^. Seasonal changes of CH_4_ and Chl-*a* in individual lakes do not support a strong link between CH_4_ and Chl-*a* concentrations^23^.

Considering lateral dispersion of CH_4_ and emissions to the atmosphere DelSontro *et al*.^28^ compared steady state CH_4_ concentration distributions with the observed decrease of CH_4_ from shallow to open water zone. They did not consider fluxes from sediments and therefore could not explain the cause for the increased CH_4_ concentrations in the littoral zone. According to DelSontro *et al*.^28^ net-oxidation is required in 30% and net-production in 70% of their lakes to reproduce the observed CH_4_ concentration distributions. However, because atmospheric fluxes of CH_4_ were calculated using *v*_*gas*_ of CO_2_ at 20 °C^28^, emissions were underestimated by ~11% in their warmest and overestimated by ~25% in their coldest lakes, respectively, affecting the reliability of the estimated net-production and net-oxidation rates of CH_4_. Furthermore, the conclusions on net-production and net-oxidation are very sensitive to lateral transport. The model of DelSontro *et al*.^28^ underestimates the transport of CH_4_ from shallow to open water zones because it uses a horizontal dispersion coefficient that underestimates lateral transport in the near boundary region^37^. Additionally, advective transport may further enhance the CH_4_ transport to the open water. Underestimation of CH_4_ transport to the open water leads in the model of DelSontro *et al*.^28^ to an underestimation of net-oxidation and overestimation of net-production of CH_4_ in the open water.

The sensitivity to lateral transport in the assessment of net-production of CH_4_ and of boundary effects prevalent in mesocosm experiments can be avoided by using a mass balance approach considering entire lake basins. According to Donis *et al*.^30^ substantial methanogenesis in oxic waters is required to close the mass balance of CH_4_ in the 5 m thick mixed surface layer of Lake Hallwil. They estimated that oxic CH_4_ production contributes ~91% of the total emissions and produces 22 time more CH_4_ than is supplied by the diffusive flux from the sediments in the mixed surface layer. However, in their mass balance Donis *et al*.^30^ underestimated the total flux of CH_4_ from littoral sediments by more than an order of magnitude and also overestimated the CH_4_ emissions to the atmosphere. Donis *et al*.^30^ apparently used 0.1225 km^2^ as value for the area of the shallow water zone in the surface mixed layer, which is ~6 times smaller than the 0.711 km^2^ suggested by the published hypsography of Lake Hallwil^40^. Furthermore, the pore-water concentrations in the top 3 cm of the sediment measured by Donis *et al*.^30^ suggest a concentration gradient of 3.4 10^4^ mmol m^−4^, which is ~1.7 times larger than the gradient used by Donis *et al*.^30^ (Supplementary Fig. S8 in Supplementary Section S3). As consequence, Donis *et al*.^30^ underestimated the diffusive sediment flux by a factor of 1.7. Re-analysis of sediment flux and emissions from Lake Hallwil (Supplementary Section S3) reveals, that the total source of CH_4_ due to diffusive sediment fluxes is slightly larger than the total loss of CH_4_ due to diffusive emissions to the atmosphere confirming that diffusive sediment fluxes are sufficient to compensate emissions to the atmosphere. This disproves that a large additional source of CH_4_ is required to close the mass balance in Lake Hallwil, i.e. the central argument of Donis *et al*.^30^ for substantial CH_4_ production in the open water. Interestingly, the ^13^C isotopic composition of CH_4_ in the open water and in the pore water at the surface of the sediments in the shallow water zone were essentially the same^30^, suggesting that the CH_4_ in the pore water near the sediment surface is the source of the CH_4_ in the open water rather than an “unknown production process(es)”^30^ generating substantial amounts of CH_4_ in oxic waters.

In the mass balance calculation uncertainty arises from the estimated loss of CH_4_ due to diffusive emissions to the atmosphere. The larger the CH_4_ emissions to the atmosphere the larger the required source of CH_4_, i.e. in our model the diffusive flux from the sediments in the shallow water. The calculations of the atmospheric CH_4_ emissions require estimates of the gas transfer velocity. Several empirical equations have been proposed to relate *v*_*gas*_ to wind speed^20,41–43^. For wind speeds typical for the systems studied here (~2 m s^−1^) the different equations provide smaller^41^, similar^42^ and, depending on the surface buoyancy flux, similar and larger values^43^ of *v*_*gas*_ than the equation of Cole and Caraco^20^ that was used here. We therefore have performed a sensitivity analysis on the implication of choosing different models for the gas transfer velocity on the results on *F*_*sed,S*_ (see Supplementary Section S4). Independent of the model chosen *F*_*sed,S*_ required to compensate total emissions to the atmosphere at 20 °C is on average smaller than the observed sediment flux *F*_*sed,Hal*_ in the mixed layer of Lake Hallwil.

In summary, our results indicate that the CH_4_ mass balances in many lakes and reservoirs do not support the conclusion that oxic methanogenesis is required to compensate CH_4_ emissions to the atmosphere. In contrast, field data and modelling results suggest that reasonable CH_4_ fluxes from sediments in shallow waters are sufficient to explain diffusive CH_4_ emissions from lakes and reservoirs, and also explain the seasonal changes in CH_4_ concentrations and CH_4_ distributions in their surface waters.

## Methods

### Data

The data set employed in this study includes numerous well resolved spatial distributions of CH_4_ measured at several times during a season in different years and in 10 different lake basins and reservoirs of different morphometry. The data set also includes seasonally resolved time series of CH_4_ measured at the central station in several of these lakes. Additionally, a seasonally resolved data set consisting of 14 transects collected during two consecutive years is available for one of the lakes (Lake Uberlingen). In total, the data set is based on 1346 individual measurements of CH_4_ concentrations in surface waters. Surface water temperatures are available for all measurements and in several lakes also continuously for several years. Wind speeds were determined from the COSMO-2 wind field^44^ available continuously for several years for all lakes. In case of the reservoirs, wind data from nearby weather stations were used. Parts of the data are discussed in^23^, detailed information on all data and systems studied is provided in Supplementary Section S1.

### Model

The interpretation of the data is supported by a model that allows dynamic simulation of a simplified mass balance of CH_4_ within the surface mixed layer of lakes and reservoirs. The model simulates CH_4_ concentrations in the surface mixed layer considering diffusive CH_4_ fluxes from sediments in the shallow water zone, diffusive gas exchange of CH_4_ with the atmosphere and horizontal mixing. Temperature dependence of diffusive gas exchange and sediment fluxes is also included. The surface layer is assumed to be fully mixed in the vertical and the CH_4_ concentrations are therefore vertically homogeneous within the surface mixed layer. Vertical transport of CH_4_ across the thermocline is neglected. In the horizontal dimension CH_4_ concentrations vary because CH_4_ is introduced from the sediments of the shallow water zone into the water column and is transported laterally by turbulent mixing.

In the simulations of entire lake basins we assume that the surface mixed layer is radially symmetric in the horizontal. The surface areas of the radially symmetric basins correspond to the true surface area of the respective basin. Because basins, sources and sinks of CH_4_ are radially symmetric, the development of the CH_4_ concentrations can be described based on the radial distance *r* from the basin center:

1a $$\frac{\partial C(r,t)}{\partial t}={K}_{h,disp}\cdot \frac{1}{H(r)\cdot r}\frac{\partial }{\partial r}(H(r)\cdot r\cdot \frac{\partial C(r,t)}{\partial r})+\frac{1}{H(r)}{F}_{sed}(r,t)-\frac{1}{H(r)}{F}_{atm}(r,t)+P(r,t)$$

1b $$\begin{array}{c}{F}_{sed}(r,t)=\{\begin{array}{cc}{F}_{sed,S}(T(t)) & r\ge {r}_{S}\\ 0 & r < {r}_{S}\end{array}\\ {F}_{atm}(r,t)={v}_{gas}(T(t),WS(t))\cdot (C(r,t)-{C}_{eq}(T(t)))\end{array}$$

The four terms on the right hand side of equ. 1a describe (i) the change of the *CH*_*4*_ concentration *C*(*r*, *t*) with time due to lateral transport, (ii) the source of CH_4_ due to the diffusive flux from the sediments, (iii) the loss of CH_4_ due to gas exchange with the atmosphere, and (iv) net-production of CH_4_, respectively. To test our hypothesis that diffusive sediment fluxes are sufficient to compensate emissions to the atmosphere, i.e. that net-production is not required to close the mass balance, we simulate the CH_4_ concentrations assuming no net-production, i.e. *P(r*, *t)* = 0.

*C*(*r*, *t*) is the concentration of CH_4_ as function of *r* and time *t*, *K*_*h,disp*_ the effective horizontal dispersion coefficient, and *H*(*r*) the spatially varying thickness of the surface layer. In the open water *H*(*r*) is equal to the mixed layer depth. Within the shallow water zone *H*(*r*) decreases linearly with *r* from the mixed layer depth to zero at the shore, i.e. at the maximum radius *r*_*max*_. *F*_*sed*_(*r*, *t*) is the diffusive flux of CH_4_ from sediments, which is zero in the open water and *F*_*sed,S*_ in the shallow water zone. *F*_*sed,S*_ depends on water temperature *T*(*t*). *F*_*atm*_(*r*, *t*) is the diffusive flux of CH_4_ to the atmosphere, *v*_*gas*_ the gas transfer velocity that depends on *T*(*t*) and wind speed *WS*(*t*), and *C*_*eq*_ the equilibrium concentration of atmospheric CH_4_ at *T*(*t*). *r*_*max*_ is the maximum radius, ${r}_{\max }=\sqrt{{A}_{surf}/\pi }$, and radius *r*_*s*_ is the distance from the center of the lake to the boundary of its shallow water zone, ${r}_{S}=\sqrt{({A}_{surf}-{A}_{S})/\pi }$. *A*_*surf*_ is the total surface area and *A*_*S*_ the surface area of the shallow water zone of the different lakes. At the boundaries horizontal fluxes are zero which implies that d*C*/d*r* = 0 at *r* = 0 and at *r* = *r*_max_.

If diffusive fluxes from the sediments are the predominant source of CH_4_ in the surface water of lakes one expects higher concentrations in the shallow water than in the open water zone^23^. The difference between surface concentrations in shallow and open water zones depends on the rate at which CH_4_ is mixed in the horizontal dimension^28^. The rate of horizontal dispersion increases with increasing length scale *L*^37,45,46^ and is described in the model by *K*_*h,disp*_. Adopting the empirical relation by Lawrence *et al*.^37^ and considering as relevant length scale the radius of the different basins *K*_*h,disp*_ = 3.2·10^−4^·*r*_*max*_^1.10^ (m^2^ s^−1^), whereby *r*_*max*_ is in m.

In addition to the radially symmetric model for investigations considering entire basins we employ a model of a vertically mixed rectangular basin for the simulation of the seasonal development of CH_4_ concentrations in the surface mixed layer along the transect in Lake Uberlingen. We assume homogeneous conditions in cross-transect direction and in the vertical dimension. Hence, the model can be condensed to a one dimensional mass balance model using coordinate x in along-transect direction (see Supplementary equs S2a,b in Supplementary Section S2). The spatially varying thickness of the surface mixed layer *H*(*x*) is given by the minimum of local water depth and *H*_*S*_. The latter is the surface mixed layer depth in the open water. The shallow water zone is defined as the region in which *H*(*x*) < *H*_*S*_.

In the transect model the horizontal dispersion coefficient *K*_*h,disp*_ is not calculated from the empirical relation of Lawrence *et al*.^37^ as in the radially symmetric model, because the choice of the length scale of dispersion is rather ambiguous considering the limited extent of the model domain in along-transect and the unlimited extent in cross-transect direction. *K*_*h,disp*_ is therefore determined by inverse modelling which provides a value of *K*_*h,disp*_ that incorporates all effects of lateral transport.

The model is implemented in MATLAB. For further details on model assumptions, parametrization and numerical solution see Supplementary Section S2.

### Analyses utilizing data and model

The models and inverse modelling techniques provide a basis for the determination of the diffusive flux of CH_4_ from the sediments, *F*_*sed,S*_, required to compensate the total emission of CH_4_ to the atmosphere, *E*_*atm*_, in different lakes and reservoirs during different seasons. *F*_*sed,S*_ can be estimated assuming steady state conditions for the respective measuring campaign. Steady state requires that *F*_*sed,S*_, *v*_*gas*_ and *C*_*eq*_ are constant in time. At steady state the total CH_4_ emission to the atmosphere is equal to the total flux of CH_4_ from the sediments of the shallow water zone:

2 $${\iint }_{Lake}{F}_{atm}dA={\iint }_{Lake}{F}_{sed}dA={\iint }_{Shallow}{F}_{sed,S}dA$$

Assuming that temperature and wind speed are horizontally homogeneous

*F*_*sed,S*_ = $\bar{{F}_{{atm}}}$·*A*_*Surf*_ /*A*_*S*_ and the spatially averaged flux to the atmosphere

$\bar{{F}_{{atm}}}={v}_{{gas}}(\bar{C}-{C}_{{eq}})$ can be calculated from the spatially averaged CH_4_ concentration $\bar{C}$.

Note that this conclusion is valid in general and does not require assumptions on the morphometry of the aquatic system. We have applied this steady state approach to estimate *F*_*sed,S*_ except in the simulations of the transect of Lake Uberlingen.

The temperature dependence of *F*_*sed,S*_ was analyzed using linear regression assuming Boltzmann-Arrhenius law:

3 $$\mathrm{ln}\,{F}_{sed,S}=C-{E}_{a}\frac{1}{{k}_{B}{T}_{a}}$$

and *C* is a constant, *E*_*a*_ the apparent activation energy, *k*_*B*_ the Boltzmann constant, and *T*_*a*_ the absolute temperature. In addition, we tested an exponential temperature dependence of *F*_*sed,S*_.

The model was applied to simulate steady state distributions of CH_4_ in the simulations considering entire basins. Utilizing equ. 1 with the estimated sediment fluxes, concentration differences between shallow and open water zones in different basins and times of the year, e.g. at different water temperatures, were simulated and compared to observations.

The capabilities of the model approach with respect to predicting seasonal changes in the CH_4_ concentrations and seasonal differences between CH_4_ concentrations in shallow and open water zones is demonstrated by the dynamic simulation of the temporal development of the CH_4_ concentrations along the cross-shore transect in Lake Uberlingen. As model domain a rectangular basin extending from shore to shore along the measured transect was used. Model results are evaluated at the times and the locations along the transect for which measurements exist. Three time-constant parameters, i.e. the activation energy *E*_*a*_ and the exponent of the pre-scaling factor of the Boltzmann-Arrhenius law describing *F*_*sed,S*_ (equ. 3), and *K*_*h,disp*_, were determined by inverse modelling of the data The comparison of model results and data is based on averaged concentrations in four distance ranges from shore (D1: <100 m, D2: 100–300 m, D3: 300–1000 m, D4: 1000–1850 m). Data are available from 14 dates during two seasons providing 56 data points for the fitting of the 3 parameters.

### Statistics

Linear regression analysis was performed using the routine “fitlm” of Matlab. In case of the assessment of temperature dependences, the logarithms of *F*_*sed*_ or of CH_4_ concentrations were used as dependent variables. Model performance was tested by regression of observed versus simulated values. The explained variance is denoted by *R*^2^ and the degrees of freedom by *df*. Two-tailed t-tests are employed to provide *p*-values testing whether slope and intercept of the regression line differ from zero (*p*) and whether the slope differs from 1 (*p*_1_).

### Re-analysis of data from Donis *et al*

We re-analyzed the data of Donis *et al*.^30^ with respect to the diffusive flux from the sediments and the atmospheric emissions of CH_4_ in Lake Hallwil. The diffusive flux from the sediments in Lake Hallwil, *F*_*sed,Hal*_, was determined assuming molecular diffusion of CH_4_ within the sediment and by using data on pore-water concentrations of CH_4_ measured in the sediment core collected on 29^th^ September 2016 from 3 m water depth in Lake Hallwil (see Fig. 5a in^30^ and Supplementary Section S3 Fig. S8). We used the same approach and parameterization as Donis *et al*.^30^ but estimated the near-surface gradient of CH_4_ in the pore water from linear regression (see Supplementary Fig. S8 in Supplementary Section S3). Pore-water concentrations were available from the sediment surface down to 3 cm depth and from depths of 7 cm and larger. Linear regression was applied to the uppermost three measurements of the pore water concentration (0, 2, and 3 cm depth). The data are very well represented by the regression line (see Supplementary α. S8 in Supplementary Section S3), suggesting that the slope of the regression line is a good estimator of the pore-water concentration-gradient near the sediment surface.

The diffusive flux to the atmosphere was calculated using several models for the gas transfer velocity^41–43,47,48^ assuming a surface water CH_4_ concentration of 0.3 mmol m^−3^ (June 2016^30^ and average concentration April to August 2016^30^), a water temperature of 20 °C (June 2016^30^), and hourly wind speeds available from station Mosen (MeteoSwiss) located at ~0.5 km distance from the shore of Lake Hallwil.

Published hypsographic data of Lake Hallwil^40^ were used to calculate the total source of CH_4_ in the surface mixed layer due to the diffusive flux from sediments and the total loss from the lake surface due to diffusive emissions to the atmosphere.

For further details see Supplementary Section S3.

## Electronic supplementary material

Supplementary information
