# Supplementary material for: Sediment fluxes rather than oxic methanogenesis explain diffusive CH4 emissions from lakes and reservoirs
Source: Sci Rep. 2019 Jan 18;9:243. doi: 10.1038/s41598-018-36530-w (PMC6338738; doi:10.1038/s41598-018-36530-w)
Supplement: Supplementary file 1 — Supplementary information [file 41598_2018_36530_MOESM1_ESM.pdf]

Supplementary Information

"Sediment fluxes rather than oxic methanogenesis explain diffusive CH_4 emissions from lakes and reservoirs"

by Frank Peeters*, Jorge Encinas Fernandez, Hilmar Hofmann

The supplementary information consists of five parts presented on a total of 26 pages:

Section S1: Information on investigated systems, measurements and data (Pg: 2-12)

Section S2: Information on models, model parameterization and numerical implementation (Pg: 13-17)

Section S3: Balance of CH_4 in Lake Hallwil: Re-analysis of the data of Donis et al. [1] (Pg: 18-22

S3.1: CH_4 flux from the sediment at 3 m water depth in Lake Hallwil (Pg: 18-20)

S3.2: CH_4 emissions from the surface waters of Lake Hallwil to the atmosphere (Pg: 20-21)

S3.3: Conclusion from the re-analysis of the data of Donis et al.[1] (Pg: 22-22)

Section S4: Sensitivity of the estimated sediment fluxes $F_{Sed,S}$ to the choice of the empirical relation for the wind dependence of the gas transfer velocity (Pg: 23-24)

Section S5: References cited in the supplement (Pg: 25-26)

Section S1: Information on investigated systems, measurements and data

All methane samples were measured using the head space technique and gas chromatography. Details on sampling and analysis are described in Hofmann et al. [2]. In the field water samples of ~ 60mL were injected with a syringe into serum bottles (volume ~122 mL) that contained 60 mL oversaturated solution of sodium chloride and were evacuated before the field work. Within the serum bottle ~100% methane is degassed from the liquid phase into the remaining headspace (22 mL). The methane concentration in the headspace was measured by gas chromatography using a flame ionization detector (GC-FID, Carlo Erba Instru- ments—GC 6000). The variation of concentrations in replicate samples was on average ~5%.

For details on the CH_4 distributions measured in the lakes Königseggsee, Illmensee, Lower Lake Konstanz (LLC), Lake Ammer, and Lake Uberlingen see Encinas Fernadez et al. [3]. Below we summarize the most important properties of the systems investigated and the data relevant to this study. We also include information on Schwarzenbach reservoir and Großer Brombachsee.

Section S1.1 Overview on the systems studied

Characteristic properties of the different systems investigated are provided in Table S1.

Table S1: Properties of the lakes and reservoirs investigated.

Lake/Reservoir Name	Area of lake surface (km^2)	Area of shallow water zone (km^2)	Maximum depth (m)	Height above sea level (m)	Total phosphorus ($\mu g\ L^{-1}$)
Königseggsee	0.16	0.06	10	627	13
Illmensee	0.64	0.16	17	692	32
Mindelsee	1.05	0.29	14	409	9
LLC (North basin	13.44	6.18	19	395	12
LLC (Central basin)	16.92	6.13	22	395	13
LLC (South basin)	31.46	12.56	45	395	8
Lake Ammer	45.99	6.91	81	533	8
Lake Uberlingen	64.69	6.00	147	395	10
Schwarzenbach	0.54	0.08	60	669	22
Großer Brombachsee	8.04	1.63	33	410	27

These systems are all lake basins except for the reservoirs Schwarzenbach and Großer Brombachsee. Schwarzenbach reservoir was built in 1926. Because it is mainly used for power generation the water level shows large fluctuations with drying out and flooding of sediments in the shallow water zone. Schwarzenbach reservoir was emptied and refilled in 1997. The reservoir Großer Brombachsee was completed in 1999, is mainly used for water storage and water flow control, and typically has a seasonal drawdown of the water level by ~5 m.

Section S1.2 Sampling locations in the systems investigated

The following figures indicate the locations of sampling stations on the lakes and reservoirs studied.

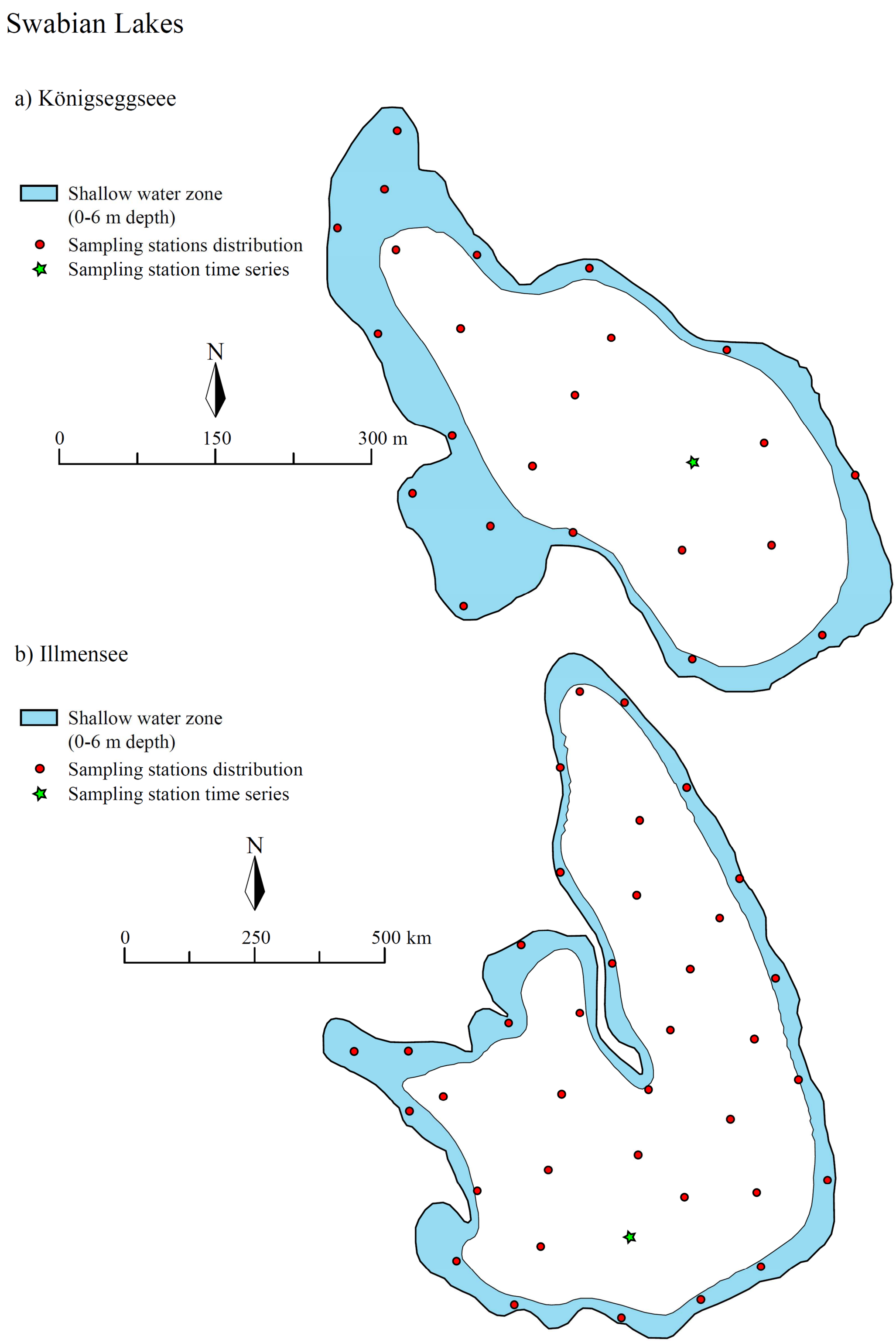

Figure S1: Sampling stations in Swabian lakes Königseggsee and Illmensee.

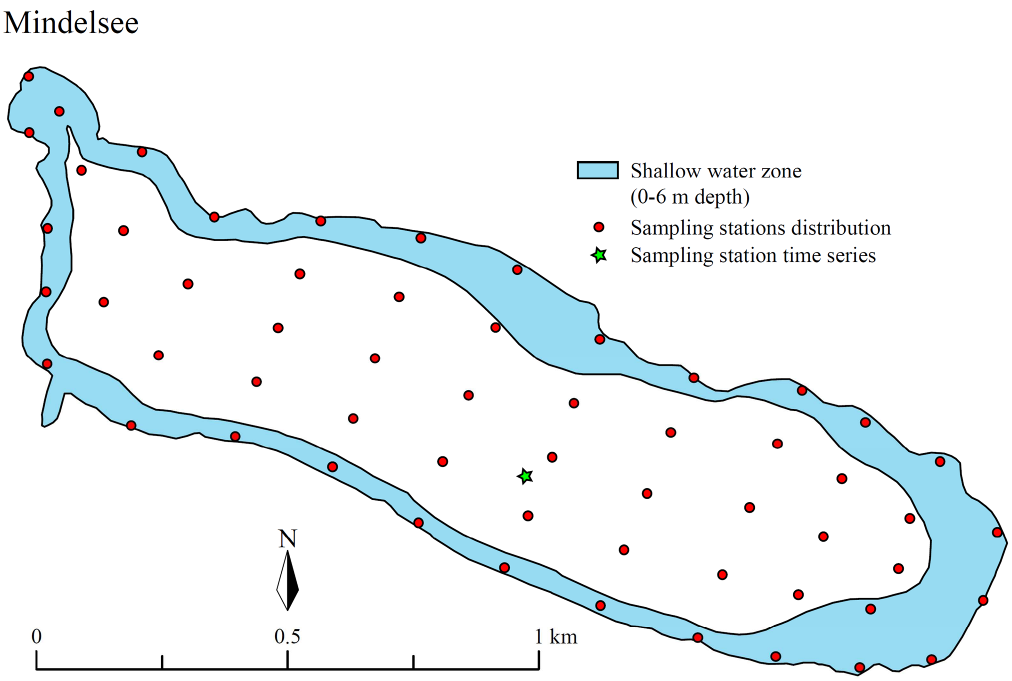

Figure S2: Sampling stations in Mindelsee.

Lower Lake Constance (LLC)

a) LLC (North basin)

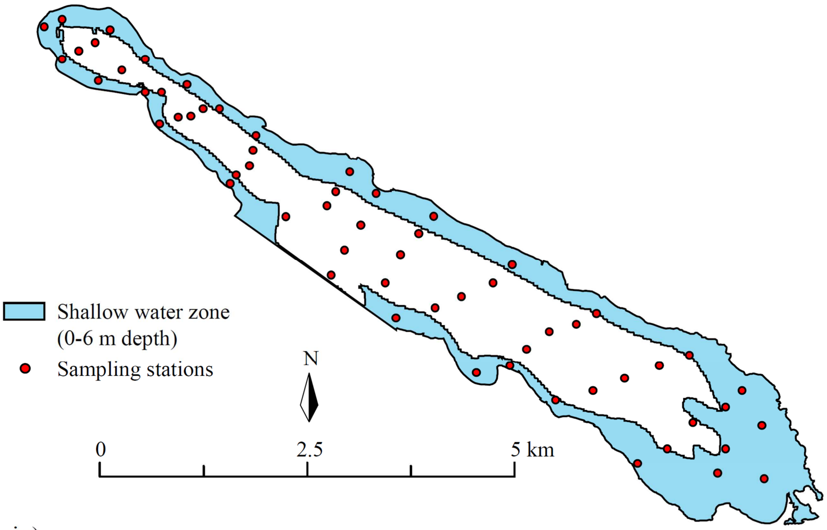

b) LLC (Central basin)

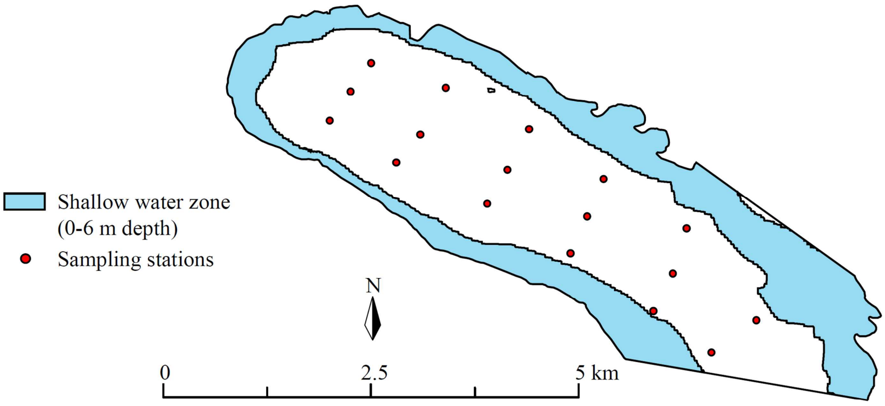

c) LLC (South basin)

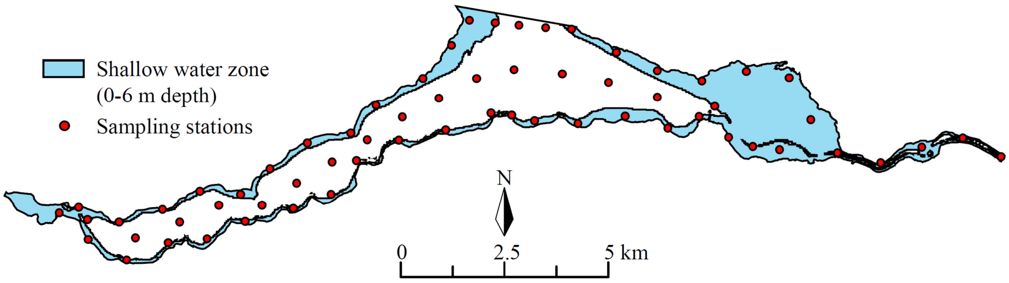

Figure S3: Sampling stations in the different basins of Lower Lake Constance: LLC (North basin), LLC (Center basin), and LLC (South basin).

Lake Ammer

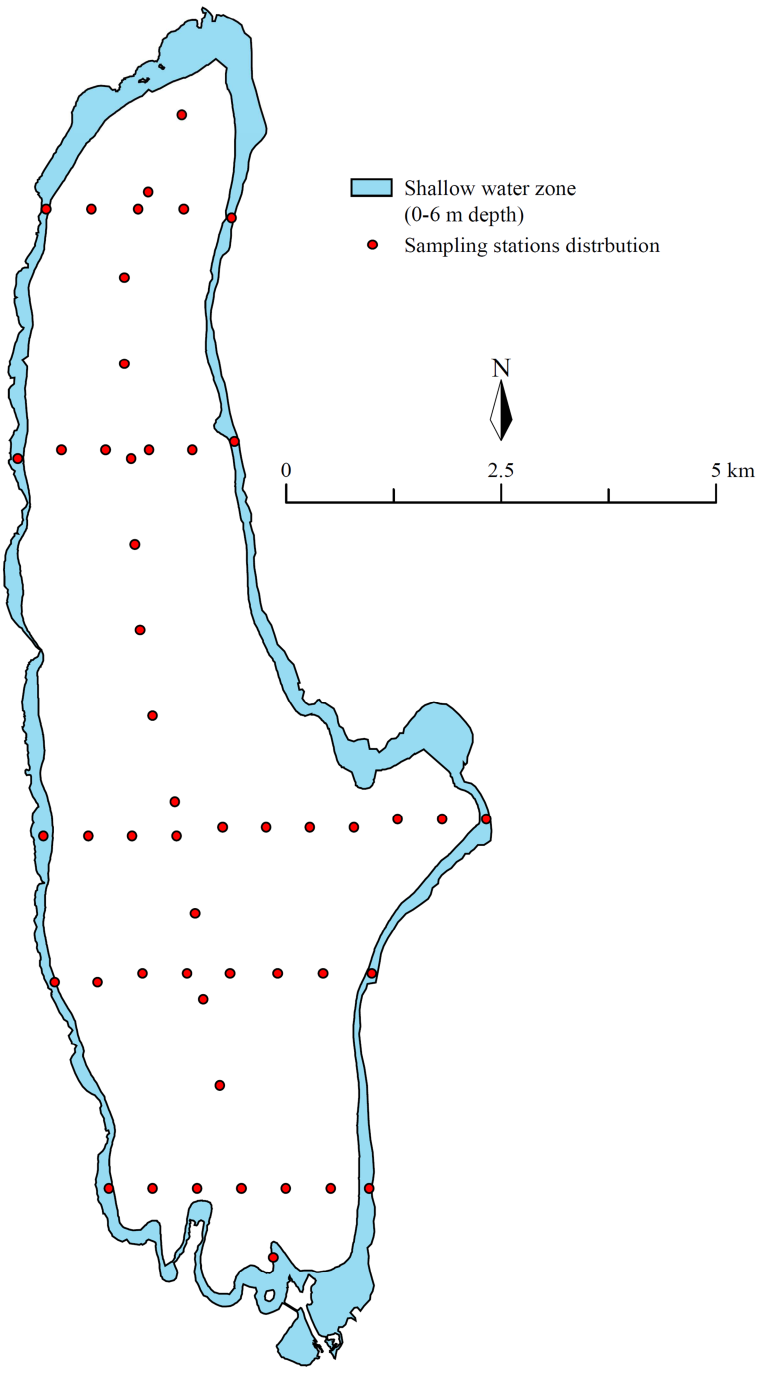

Figure S4: Sampling stations in Lake Ammer.

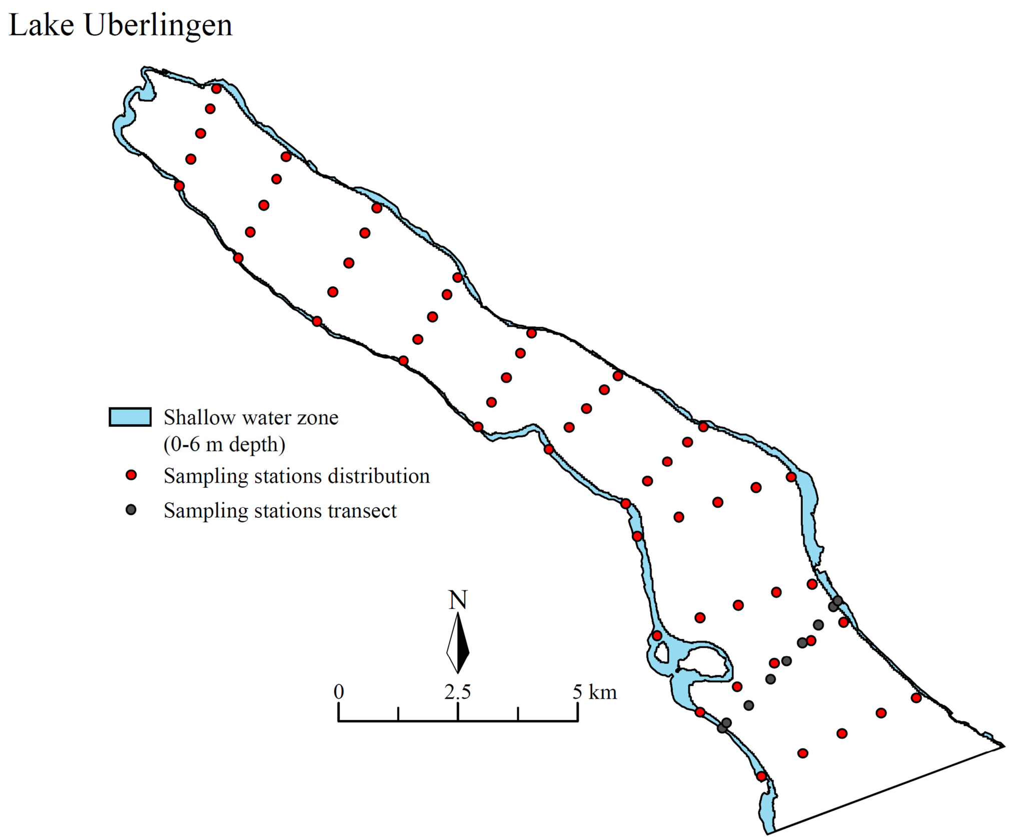

Figure S5: Sampling stations in Lake Uberlingen.

Reservoirs

a) Schwarzenbach reservoir

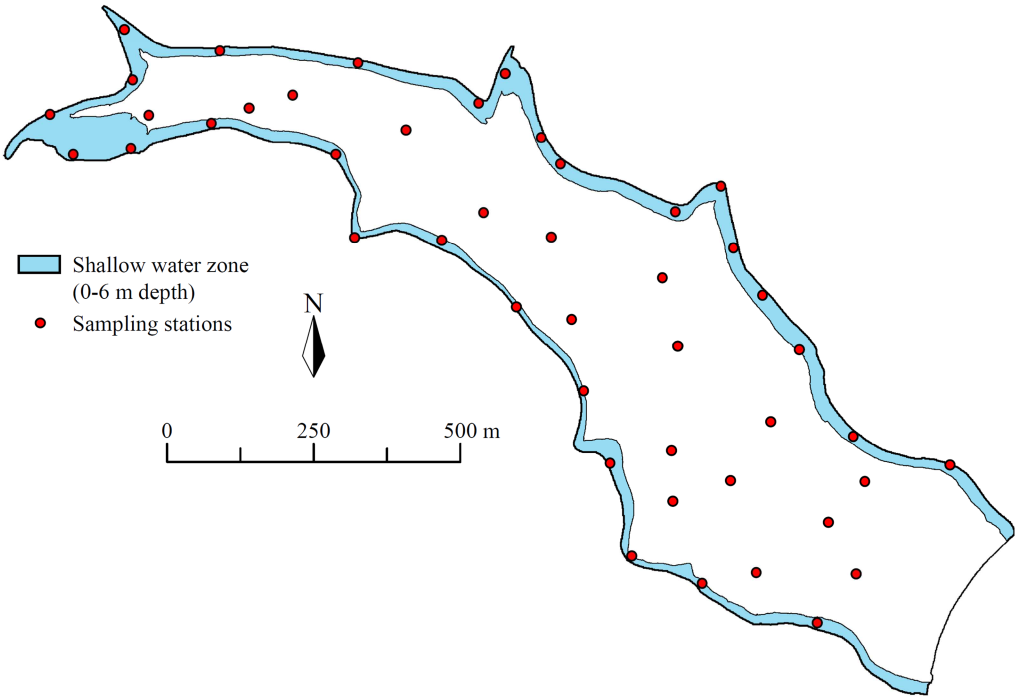

b) Großer Brombachsee

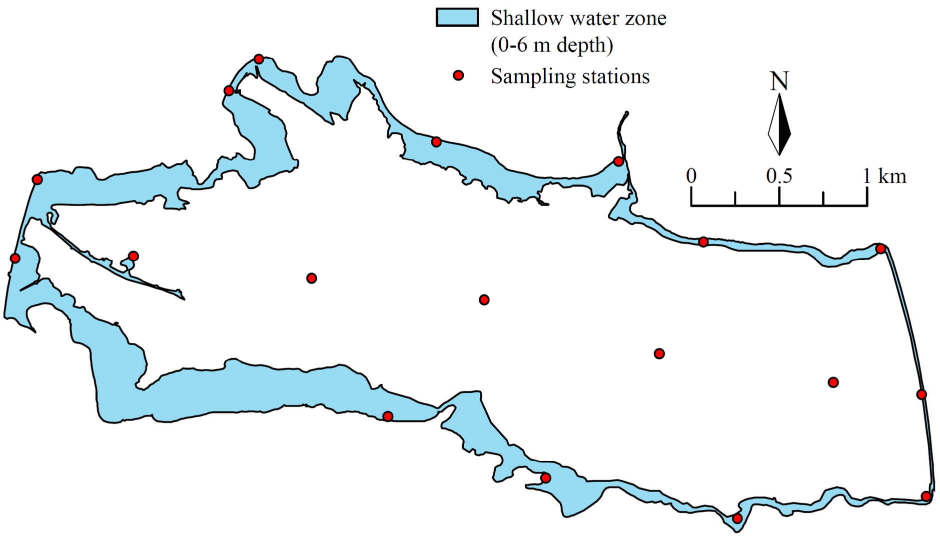

Figure S6: Sampling stations in the reservoirs Schwarzenbach and Großer Brombachsee.

The number of surface measurements collected during the different campaigns in the different lakes is listed in Table S2. These surface measurements were used to calculate the average surface concentration of CH_4 in the entire basin and in the littoral for the different campaigns.

Table S2: Number of sampling points collected in the campaigns measuring CH_4 distributions. Except for Schwarzenbach reservoir and Großer Brombachsee the data are taken from Encinas Fernadez et al. [3]

Lake	Date of campaign	Number of surface measurements	Average CH_4 concentration in the entire basin (mmol m^{-3})	Average CH_4 concentration in the littoral (mmol m^{-3})
Königseggsee	28.09.2012	24	0.596	0.672
	14.11.2012	24	0.153	0.112
	03.05.2013	24	0.371	0.424
	18.07.2013	24	0.533	0.565
	17.10.2013	24	0.617	0.674
	27.05.2014	24	0.277	0.281
	25.06.2014	24	2.034	2.176
Illmensee	26.09.2012	36	0.651	0.667
	16.11.2012	36	1.073	0.850
	07.05.2013	36	0.356	0.454
	15.07.2013	38	0.828	0.878
	17.10.2013	38	0.886	0.789
	10.12.2013	37	0.111	0.123
	28.03.2014	38	0.178	0.372
	25.06.2014	38	1.471	1.718
Mindelsee	10.08.2012	58	0.894	1.204
LLC (North basin)	19.04.2011	31	1.628	1.414
	10.05.2011	32	3.748	5.287
	07.06.2011	50	3.687	4.424
LLC (Central basin)	19.04.2011	26	1.199	1.175
	10.05.2011	26	4.528	5.673
	07.06.2011	33	1.505	2.340
LLC (South basin)	19.04.2011	62	0.622	1.294
	10.05.2011	63	2.185	4.426
Lake Ammer	27.06.2011	50	0.290	0.400
Lake Uberlingen	07.09.2012	55	0.170	0.540
Schwarzenbach reservoir	14.04.2016	45	0.111	0.166
	12.05.2016	45	0.142	0.143
	23.06.2016	45	0.099	0.110
	18.08.2016	45	0.180	0.342
Großer Brombachsee	27.09.2017	18	0.599	0.597

In Königseggsee, Illmensee, and Mindelsee additional surface samples were taken at the central station (indicated by a star in the figures above) at different times during the season and in different years: 10 samples in Königseggsee, 10 samples in Illmensee, and 30 samples in Mindelsee.

Section S1.3 Cross-shore transect in Lake Uberlingen

The sampling points along the cross-shore transect in Lake Uberlingen are depicted in Fig. S7.

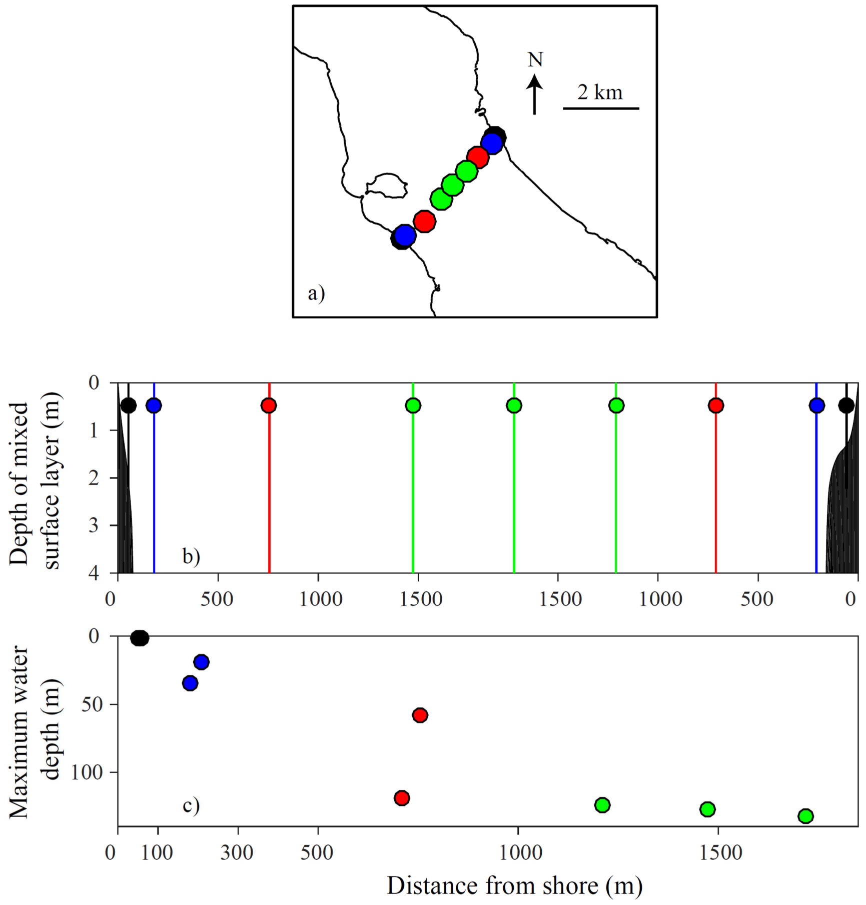

Figure S7: Sampling points along the cross-shore transect in Lake Uberlingen. (a) Sampling points (symbols) and shore line. (b) Sampling points along the cross-shore transect in the surface mixed layer. Basin boundaries are indicated by the black areas. (c) Sampling points as function of distance to shore and local maximum water depth. Sampling points with the same color belong to the same distance range. Distance ranges are D1: 0-100 m (black); D2: 100-300 m (blue); D3: 300-1000 m (red); D4: 1000 - 1850 m (green).

The CH_4 concentrations along the transect were measured 14 times during different times of the season in 2009, 2010 and 2011. In total 123 surface CH_4 concentrations are available from the different stations along the transect (Table S3).

Table S3: CH_4 concentrations along the transect in Lake Überlingen. Missing measurements are indicated by NaN.

Date	Surface methane concentration (mmol L^{-1})								
dd-mm-yyyy	St1	St2	St3	St4	St5	St6	St7	St8	St9
13-05-2009	0.233	0.175	0.095	0.125	0.096	0.119	0.100	0.151	0.291
27-05-2009	0.525	0.573	0.274	0.392	0.293	0.312	0.404	0.276	0.763
06-07-2009	0.833	0.564	0.423	0.370	0.242	0.368	0.296	0.352	0.826
03-08-2009	0.279	NaN	0.239	0.227	0.185	0.293	0.276	0.592	1.364
02-09-2009	0.551	0.364	0.289	0.222	0.204	0.251	0.241	0.293	1.178
23-10-2009	0.257	0.178	0.115	0.132	0.118	NaN	0.078	0.109	0.177
07-04-2010	0.191	0.154	0.081	0.038	0.087	0.096	0.065	0.106	0.118
10-05-2010	0.203	0.190	0.096	0.151	0.153	0.133	0.132	0.144	0.706
09-06-2010	0.288	0.290	0.183	0.168	0.239	0.199	0.204	0.351	0.542
12-07-2010	0.808	1.005	0.262	0.314	0.157	0.138	0.169	0.247	0.056
11-08-2010	0.527	0.415	0.180	0.167	0.132	0.132	0.129	0.299	0.735
22-09-2010	0.415	0.178	0.117	0.103	0.129	0.123	0.096	0.099	0.347
25-10-2010	0.102	0.092	0.067	0.037	0.104	0.097	0.073	0.088	NaN
28-02-2011	0.072	0.065	0.085	0.058	0.052	0.117	0.053	0.083	0.242
Distance from southern shore along the transect (m)									
	52	180	756	1472	1978	2486	2985	3487	3637
Water column depth at the station (m)									
	1	34	58	128	132	125	119	20	1

Section S2: Information on models, model parameterization and numerical implementation

The CH_4 concentrations in the surface mixed layer are simulated considering loss of CH_4 due to emissions from the lake surface to the atmosphere and gain of CH_4 due to the flux of CH_4 from the sediments into the surface mixed layer. The surface mixed layer is assumed to be mixed fully in the vertical and the CH_4 concentrations are therefore homogeneous in the vertical dimension. In the horizontal dimension CH_4 concentrations vary because CH_4 is introduced from the sediments of the shallow water zone into the water column. The model considers lateral transport by turbulent diffusion which reduces lateral gradients.

In the simulations of entire lake basins we assume that the surface mixed layer is radially symmetric in the horizontal dimension. The surface areas of the radially symmetric basins correspond to the true surface area of the respective basin. Because basins, sources and sinks of CH_4 are radially symmetric and CH_4 concentrations in the surface mixed layer are independent of depth, the CH_4 concentrations only depend on the radial distance r from the basin center and on time. Because CH_4 concentrations are a function of r, also the flux of CH_4 from the lake surface F_{atm} depends on r. The model of the mass balance of methane in the surface mixed layer assuming radially symmetric systems reads:

$$\frac{\partial C(r,t)}{\partial t} = K_{h,disp} \cdot \frac{1}{H(r)\cdot r}\frac{\partial}{\partial r}\left(H(r)\cdot r\cdot\frac{\partial C(r,t)}{\partial r}\right) + \frac{1}{H(r)}F_{sed}(r,t) - \frac{1}{H(r)}F_{atm}(r,t) + P(r,t) \qquad \text{S1a}$$

$$F_{sed}(r,t) = \begin{cases} F_{sed,S}(T(t)) & r \ge r_S \\ 0 & r < r_S \end{cases} \qquad \text{S1b}$$

$$F_{atm}(r,t) = v_{gas}(T(t),WS(t))\cdot\left(C(r,t) - C_{eq}(T(t))\right)$$

The four terms on the right hand side of S1a describe (i) the change of $C(r,t)$ with time due to lateral transport, (ii) the source of CH_4 due to the flux from the sediments, (iii) the loss of CH_4 due to gas exchange with the atmosphere, and (iv) net production of CH_4, respectively. To test our hypothesis that sediment fluxes are sufficient to compensate emissions, i.e. that net production is not required to close the mass balance, we simulate the methane concentrations assuming no net production, i.e. $P(r,t) = 0$.

In equations S1a and S1b $C(r,t)$ is the concentration of CH_4 as function of r and time t, $K_{h,disp}$ the effective horizontal dispersion coefficient and $H(r)$ the spatially varying thickness of the surface layer. $F_{sed}(r,t)$ is the CH_4 flux from sediments, which is zero in the open water and $F_{sed,S}$ in the shallow water zone. $F_{sed,S}$ depends on water temperature $T(t)$. $F_{atm}(r,t)$ is the CH_4 flux to the atmosphere, v_{gas} is the gas transfer velocity that depends on $T(t)$ and wind

speed $WS(t)$, and C_{eq} is the equilibrium concentration of atmospheric methane at $T(t)$. The maximum radius of the basin $r_{\max} = \sqrt{A_{surf}/\pi}$ and the radius of the shallow water zone $r_S = \sqrt{(A_{surf} - A_S)/\pi}$ are determined from the total surface area, A_{surf}, and the surface area of the shallow water zone, A_S, of the different lake basins. At the lateral boundaries horizontal fluxes are zero which implies that $dC/dr = 0$ at $r = 0$ and $r = r_{\max}$.

In addition to the radially symmetric model for investigations considering entire basins we have used a model of a vertically mixed rectangular basin for the simulation of the seasonal development of CH_4 concentrations in the surface mixed layer along the transect in Lake Uberlingen. We have assumed homogeneous conditions in cross-transect direction and in the vertical dimension. Hence, the model can be condensed to a one dimensional mass balance model with coordinate x in along transect direction:

$$\frac{\partial C(x,t)}{\partial t} = K_{h,disp} \cdot \frac{1}{H(x)} \frac{\partial}{\partial x}\left(H(x) \cdot \frac{\partial C(x,t)}{\partial x} \right) + \frac{1}{H(x)} F_{sed}(x,t) - \frac{1}{H(x)} F_{atm}(x,t) + P(x,t) \qquad \text{S2a}$$

$$F_{sed}(x,t) = \begin{cases} F_{sed,c}(T(t)) & H(x) < H_{lit} \\ 0 & H(x) \geq H_{lit} \end{cases} \qquad \text{S2b}$$

$$F_{atm}(x,t) = v_{gas}(T(t), WS(t)) \cdot \left(C(x,t) - C_{eq}(T(t)) \right)$$

and $H(x)$ is the depth of the mixed layer as function of the coordinate x. As in the radially symmetric model the four terms on the right hand side of S2a represent (i) lateral transport by turbulent diffusion, (ii) the source of methane due to fluxes from the sediments, (iii) the loss of methane due to gas exchange with the atmosphere, and (iv) an additional source of CH_4 due to net production. The last term was set to zero, i.e. $P(x,t) = 0$, as in the radially symmetric case.

The depth of the surface mixed layer was ~6 m in all campaigns in all lakes and reservoirs during which spatial distributions were measured. In the analysis of the time series data from the center of some of these lakes we also used 6 m for the mixed layer depth for better comparability.

In the seasonal simulations of the transect in Lake Uberlingen we used a mixed layer depth of 4 m. This was the average depth of the mixed layer during the stratified seasons in 2009 and 2010 estimated using temperature profiles measured at the central station in Lake Uberlingen at 1 minute time interval with a thermistor chain consisting of temperature sensors deployed at 0.5, 0.9, 1.3, 1.7, 2.1, 2.5, 2.9, 3.4, 3.9, 4.4, 5.4, 6.4, 8.4, 10.4, 12.4, 14.4, 16.4,

18.4, 20.4 m depth and larger spacing at large depth. In 2009 we used a thermistor chain (PME: Precision Measurement Engineering) and from 25 June 2010 onwards individual TR1050 loggers (RBR Ltd., Canada) attached to a mooring. The mixed layer depth was defined as the depth at which temperature is 0.5°C lower than the temperature at 0.5 m water depth and was averaged from 1 April to 30 September in 2009 and from 25 June to 30 September in 2010. Surface temperatures measured at 0.5 m depth were available continuously over the entire time period from April 2009 until April 2011.

The gas transfer velocity of methane was calculated based on the empirical relation of Cole and Caraco [4] for the gas transfer velocity $v_{gas,600}$ of CO_2 in freshwater at 20°C (i.e. at Schmidt number Sc_{CO2} (20°C) = 600) as function of wind speed U_{10} measured 10 m above ground. We use the empirical model of Cole and Caraco [4] because it has been widely used and recent studies have shown that it agrees well with detailed observations from flux chamber measurements in lakes [5]. We have additionally investigated the sensitivity of our results with respect to the choice of the empirical relation for $v_{gas,600}$ (see SI section S4). The Schmidt number dependence of the gas transfer velocity suggested by Liss and Merlivat [6] and the Schmidt number for methane Sc_{CH4} as function of temperature [7] were utilized to calculate the gas transfer velocity of methane:

$$v_{gas,600} = 2.07 + 0.215 \cdot U_{10}^{1.7} \; cm\,h^{-1}$$

$$v_{gas} = v_{gas,600} \cdot \left(\frac{Sc_{CH4}}{600} \right)^{n} \qquad n = \begin{cases} -2/3 & if \;\; U_{10} \le 3.7\,\mathrm{ms}^{-1} \\ -1/2 & if \;\; U_{10} > 3.7\,\mathrm{ms}^{-1} \end{cases} \qquad \text{S3}$$

As in the other studies investigating the relative importance of oxic methane production, e.g. Bogart et al. [8], DelSontro et al. [9], and Donis et al. [1], we do not consider spatial variability of the gas transfer velocity. Gas transfer velocities can vary in space and the variability may also depend on lake morphometry [5,10], e.g. v_{gas} may be lower in wind sheltered regions and larger in the vicinity of inflows. The inflows may introduce additional near surface turbulence than suggested by common empirical wind speed based relations. The variability of the gas transfer velocity due to wind shelter can be particularly large in small lakes in which wind shelter by e.g. trees or steep shores affect a substantial fraction of the surface area of the lake. However, the effect of spatial variability on v_{gas} is small in lakes of $5 \cdot 10^5$ m^2 surface area and larger [5], and basin wide average v_{gas} closely agree with v_{gas} obtained from the empirical relation of Cole and Caraco [5]. In smaller lakes the empirical relation of Cole and Caraco [4] typically underestimates basin averaged v_{gas}[5]. This implies that in these

lakes $F_{sed,S}$ calculated from equ. S3 are an upper bound of the diffusive sediment fluxes required to compensate atmospheric emissions.

Note that in spite of the usage of spatially constant v_{gas} the atmospheric fluxes in the model vary in space, because the surface concentrations are higher near shore than in the open water.

The atmospheric equilibrium concentration of methane was calculated using the solubility of CH_4 [11] and the local partial pressure of CH_4. The latter was estimated assuming a molar fraction of 1.8 ppm of CH_4 in air at local air pressure calculated from altitude using the standard barometric formula.

In the simulations of full lake basins we estimated the horizontal dispersion coefficient $K_{h,disp}$ from the empirical relation of Lawrence et al [12]:

$$K_{h,disp} = 3.2\cdot 10^{-4}\cdot L^{\,1.10}\ (\mathrm{m}^2\ \mathrm{s}^{-1}) \qquad \text{S4}$$

and L is the length scale of dispersion in m. The empirical relation equ. S4 is the same as that used by DelSontro et al. [9]. It provides an estimate of the horizontal dispersion coefficient in the open water but is smaller than the dispersion coefficient obtained from experiments conducted near the lake boundaries [12]. Hence, equ. S4 can be considered providing a rough estimate of the lower limit of $K_{h,disp}$ at a basin scale, which however underestimates horizontal dispersion especially in the near boundary region.

Horizontal dispersion is length scale dependent (e.g. equ. S4 [12]; Okubo [13]). The length scale dependence is caused by the cascade of eddy sizes and also by shear dispersion (see e.g. Okubo [13], Peeters et al. [14] and numerous other studies). Nevertheless, the time scale of mixing $t_{mix} \sim L^2/K_{h,disp}$ increases with length scale of mixing, e.g. with the length-scale dependence of $K_{h,disp}$ in equ. S4 one obtains $t_{mix} \sim L^2/L^{1.1} = L^{0.9}$.

The empirical relation equ. S4 of Lawrence et al. [12] requires a length scale for the calculation of $K_{h,disp}$ and we have used the radius r_{max} of the circle with the same surface area of the basin as length scale. This length scale can be consistently applied to the different basins and has a natural connection to the size of the largest eddies in the systems. In all simulations considering entire basins we have therefore used $K_{h,disp} = 3.2\cdot 10^{-4}\cdot r_{max}^{\,1.10}$ ($\mathrm{m}^2\ \mathrm{s}^{-1}$), whereby r_{max} is in m.

In the simulation of the concentration distribution along the transect in Lake Uberlingen, the natural choice of a length scale is unclear. Using half the transect length (1850 m) would correspond to the length scale definition employed in the other systems

assuming a circular basin with radius of this length scale. However, in cross transect direction eddies of the turbulent velocity field can have larger extent than half the transect length. Hence, half the transect length most likely underestimate the appropriate length scale. Because the choice of the length scale is unclear we fitted the dispersion coefficient by inverse modelling. Note that the result of the fitting provides $K_{h,disp}$ = 1.4 m^2 s^{-1}, which is only slightly larger than the dispersion coefficient obtained from the empirical equation of Lawrence et al. [12] at half the transect length ($K_{h,disp}$ = 1.3 m^2 s^{-1} at a length scale of 1850 m). According to the empirical relation of Lawrence et al [12] $K_{h,disp}$ = 1.4 m^2 s^{-1} corresponds to a length scale of 2040 m, which is only slightly larger than half the transect length. The fitted dispersion coefficient $K_{h,disp}$ = 1.4 m^2 s^{-1} can be considered to approximate the overall effect of lateral transport including not only lateral dispersion in open and near boundary region but also all other transport leading to exchange that can be approximated by a diffusion type process.

The models were implemented in MATLAB. The partial differential equations were solved using the method of lines by discretizing the spatial coordinate and solving the resulting coupled system of ordinary differential equations using the ode15s solver of MATLAB. In the model applications simulating steady state conditions in radially symmetric basins the spatial coordinate was discretized with 1000 grid cells of equal size. Equilibrium concentration distributions were obtained by simulating 10 years with time-constant forcing. In case of the simulation of the seasonal development in Lake Uberlingen using a rectangular basin, the x-coordinate was discretized using a grid cell size of 5 m.

Section S3: Balance of CH_4 in Lake Hallwil: Re-analysis of the data of Donis et al. [1]

In the following we re-analyze the data of Donis et al. [1] and demonstrate that the main conclusion of Donis et al. [1] is not supported by their data. Donis et al. [1] argued that sediment fluxes do not provide sufficient CH_4 to compensate methane losses to the atmosphere. Specifically, they estimated that CH_4 emissions in Lake Hallwil are ~26 times larger than the total flux of CH_4 from shallow water sediments (Table 2 in Donis et al. [1]: 5040 mol d^{-1} versus 196 mol d^{-1}). They concluded that a large source of methane, i.e. net production of 4600 mol d^{-1} within the oxic surface water, is required to close the balance of CH_4. In contrast to this argument, a consistent analysis of the data of Donis et al. [1] shows that the methane introduced into the surface mixed layer from shallow water sediments agrees very well with the loss of methane due to emissions of CH_4 to the atmosphere. Hence, the data does not support the argument of Donis et al. [1] of a large missing source of methane and thus does not provide evidence for substantial oxic methane production.

The following sections provide details on our re-analysis of the data of Donis et al. [1]. We first estimate the CH_4 flux from the shallow sediments based on the pore water profile of CH_4 measured in a sediment core collected by Donis et al. [1] on 29th of September 2016 at station S1 in Lake Hallwil. The water depth at this station was 3 m and the CH_4 flux estimated from the pore water concentrations of this core was therefore used by Donis et al. [1] to represent the sediment flux into the surface mixed layer which extends from 0-5 m water depth (Donis et al. [1]). After the analysis of the sediment fluxes we present estimates on the CH_4 emissions from the surface of Lake Hallwil to the atmosphere.

S3.1. CH_4 flux from the sediment at 3 m water depth in Lake Hallwil

In the following we demonstrate that Donis et al. [1] underestimated the overall flux from littoral sediments by an order of magnitude. They underestimated the flux per unit sediment area from sediments in the shallow water zone by a factor of 1.7 because they employed a too small gradient ofthe pore water CH_4 concentration in the sediment core. Further, in their estimate of the total flux from littoral sediments they employed an area of the littoral zone that is ~ 5.8 times smaller than the area of the shallow water zone obtained from published morphometry of Lake Hallwil (Ribi et al. [15]).

S3.1.a. CH_4 Flux from sediments (CH_4 release from sediments per unit area and time) in the shallow water zone.

Donis et al. [1] estimated the CH_4 flux from the sediments of the shallow water zone based on the pore water CH_4 profile measured in a core collected on 29th September 2016 from a site

with 3 m water depth (site S1). The diffusive sediment flux was estimated from

$$F_{seed} = -\phi D_{CH4} \theta^{-2} \frac{dC_{CH4}}{\partial z} \qquad S5$$

using porosity ϕ=0.9, molecular diffusivity D_{CH4} = 1.5 10^{-9} m^2 $s^{-1}$and tortuosity θ=1.2 from Donis et al. [1].

Donis et al. [1] assumed a CH_4 pore water concentration of 1 mmol L^{-1} at 5 cm depth and a linear decrease of the pore water concentration towards the surface value of ~10^{-3} mmol L^{-1}. This implies a gradient of 2.0 10^4 mmol m^{-4}. With this gradient and equ. S5 Donis et al. [1] obtained a CH_4 flux from the sediments in the surface mixed layer of 1.6 mmol m^{-2} d^{-1} (see Pg.4 in [1]).

We have digitized the data on the pore water concentrations of Donis et al. (see Fig. 5 in [1]) and depict these data in Figure S8. The gradient assumed by Donis et al. [1] is included in red which clearly is not consistent with the data (Figure S8). Fitting a straight line to the pore water concentrations measured within the top 3 cm of the core provides an estimate of the concentration gradient of 3.4·10^4 mmol m^{-4} which is 1.7 times larger than the gradient used by Donis et al. [1]. The sediment flux estimated correctly from the CH_4 gradient in the pore water and from equ. S5 is $F_{sed,Hal}$ = 2.8 mmol m^{-2} d^{-1} and thus substantially larger than the published value of 1.6 mmol m^{-2} d^{-1}.

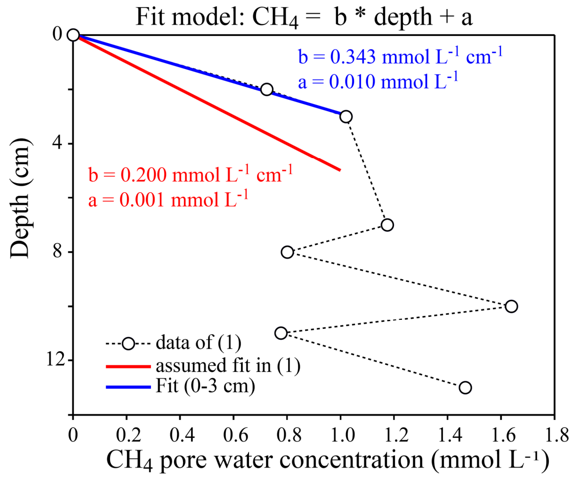

Figure S8: CH_4 pore water concentrations measured in a sediment core in Lake Hallwil collected on 29th September 2016 at 3 m water depth (digitized from Fig. 5 in Donis et al. [1]). The vertical gradient of pore water CH_4 concentration obtained using a linear fit to the data from the top 3 cm of the sediment core is 3.4 10^4 mmol m^{-4} (blue line). Donis et al. [1] used a gradient of 2.0 10^4 mmol m^{-4} (red line) in their mass balance.

S3.1.b. Total flux due to the sediment fluxes in the littoral zone

The total flux from the sediments of the littoral zone within the mixed surface layer (Zone 2: 0-5m, see Table 2 in Donis et al. [1]) can be calculated by multiplying the sediment flux per unit area with the area covered by sediments in the surface mixed layer.

Using their sediment flux of 1.6 mmol m^{-2} d^{-1} Donis et al. [1] obtain a total flux of 196 mol d^{-1} (Table 2 in [1]). This result implies that Donis et al. [1] used 0.123 km^2 as the area of the sediments in the shallow water zone (196 mol d^{-1} / 1.6 10^{-3} mol d^{-1} m^{-2} = 122500 m^2). However, according to Ribi et al. [15] the surface area of Lake Hallwil is A_{surf} = 9.947 10^6 m^2 and the cross section at 5 m depth is A_{5m}= 9.23647 10^6 m^2 which implies a sediment area of 0.711 km^2 within the surface mixed layer between 0 and 5 m depth. Hence the sediment area employed by Donis et al. [1] is ~ 5.8 times smaller than the sediment area obtained from morphometric data.

Using the correct sediment area and the sediment flux correctly estimated from the pore-water concentration measured within the top 3 cm of the sediment core one obtains in the mixed surface layer a total source of CH_4 due to sediment fluxes of: $S_{sed,total} = 2.8 \cdot 10^{-3}$ mol m^{-2} d^{-1} · 7.11 10^5 m^2 = 1990 mol d^{-1}, which is 10 times larger than the total source due to sediment fluxes of 196 mol d^{-1}used in the mass balance by Donis et al. (see Table 2 in [1])

S3.2. CH_4 emissions from the surface waters of Lake Hallwil to the atmosphere

The mass balance argument by Donis et al. [1] implicitly assumes steady state conditions. As fluxes, emissions and bio-chemical transformations of CH_4 are known to be temperature dependent [16,17], the mass balance should be performed at the same temperature. The sediment cores were taken on 29th September 2016 when water temperatures in Lake Hallwil were ~20°C (estimated from water temperatures measured at station Seengen, Kanton Aargau). Hence, the following discussion of emissions from Lake Hallwil is based on calculations for a water temperature of 20°C.

Gas exchange at the lake surface is determined from the relation:

$$F_{atm} = v_{gas}(T,WS) \cdot \left(C - C_{eq}(T)\right) \qquad \text{S6}$$

According to Donis et al. [1] the average surface water concentration of CH_4 between April and August 2016 was ~ 0.3 mmol m^{-3} (Pg. 4 in [1]). In June 2016 surface water temperatures were 20°C and the surface concentration was also ~0.3 mmol m^{-3} (see Fig. 4a in [1]). The atmospheric equilibrium concentration of CH_4 at 20°C is 0.0026 mmol m^{-3} assuming a

salinity of 0.3 g kg^{-1} and a local atmospheric pressure of 0.95 atm. The latter was estimated from the standard barometric pressure equation and the height above sea level of Lake Hallwil of 449 m. Hence, in equ. S6 the term $(C-C_{eq})$ = 0.2974 mmol m^{-3}.

The gas exchange velocity depends on wind speed and, via the Schmidt number, on the type of gas and on water temperature (equ. S3). The Schmidt number of CH_4 at T=20°C and S =0.3 g kg^{-1} is Sc_{CH4} = 616 [7]. Hourly wind speed data are available from the station Mosen located at about 1 km distance from Lake Hallwil (Swiss Federal Office for Meteorology and Climatology: MeteoSwiss). From Sc_{CH4} and the wind speed time series gas exchange velocities at 20°C were determined using the models of Cole and Caraco [4] and the relations of MacIntyre et al. [18] for positive surface buoyancy fluxes and for the combination of positive and negative buoyancy fluxes. From the gas exchange velocities averaged gas velocities were determined for the time period from April to the end of August 2016 and for June 2016 (Tabel S3). Note, that during periods of water surface heating, e.g. in June, surface buoyancy fluxes can be expected to be positive. We additionally include results on gas exchange velocities based on the relations of Guernin et al. [19], Schwarzenbach et al [20] and Crusius and Wanninkhov [21]. Using the different estimates of the gas exchange velocity we determined the fluxes to the atmosphere F_{atm} from equ. S6. The total emission of CH_4 to the atmosphere were calculated from the surface area A_{surf} = 9.947 10^6 m^2 and the fluxes F_{atm}. The results of these calculations are listed in Table S3.

Table S3: Gas exchange velocities, atmospheric fluxes F_{atm}, and total CH4 emissions to the atmosphere from Lake Hallwil 2016.

Model	April to August			June		
	v_{gas} (m d^{-1})	F_{atm} (mmol m^{-2} d^{-1})	total emission (mol d^{-1})	v_{gas} (m d^{-1})	F_{atm} (mmol m^{-2} d^{-1})	total emission (mol d^{-1})
Cole and Caraco[4]	0.63	0.187	1860	0.62	0.182	1810
MacIntyre et al. [18] (positive buoyancy flux)	0.64	0.191	1900	0.61	0.181	1800
MacIntyre et al. [18] (all buoyancy fluxes)	0.91	0.272	2706	0.87	0.259	2576
Guernin et al. [19],	0.62	0.185	1840	0.60	0.180	1790
Schwarzenbach et al.[20]	0.56	0.165	1641	0.55	0.164	1631
Crusius and Wannhinkov [21]	0.30	0.088	875	0.27	0.081	806

In all cases the emissions are substantially lower than the value of 5040 mol d^{-1} used by Donis

et al. in their mass balance (Table 2 in [1]).

S3.3. Conclusion from the re-analysis of the data of Donis et al.[1]

Based on the CH_4 pore-water concentrations measured by Donis et al. [1] in a sediment core taken at 3 m water depth, the total source of CH_4 due to diffusion from the sediments into the mixed surface layer can be estimated to be $S_{sed,total}$ ~1990 mol d^{-1}. This value is slightly larger than the total loss of CH_4 from the surface water of Lake Hallwil due to diffusive gas exchange to the atmosphere at 20°C. The gas transfer velocity model of Cole and Caraco [4] and of MacIntyre et al. [18] for periods with positive surface buoyancy flux agree very well and provide average total emissions of CH_4 at 20°C water temperature of $E_{atm,total}$ = 1810 mol d^{-1} and $E_{atm,total}$ = 1800 mol d^{-1}, respectively, for June 2016, and of $E_{atm,total}$ = 1860 mol d^{-1} and $E_{atm,total}$ = 1900 mol d^{-1}, respectively, for the period from April to August. In June 2016 surface water temperatures were ~20°C and because the lake is warming the buoyancy flux can be expected to be positive. The sediment flux $F_{sed,S}$ required to compensate atmospheric emissions in Lake Hallwil is $F_{sed,S}$ = 2.5 mmol m^{-2} d^{-1} to 2.7 mmol m^{-2} d^{-1} (1800 mol d^{-1} / 0.711 10^{6} m^{2} to 1900 mol d^{-1}/ 0.711 10^{6} m^{2}) and therefore smaller than the sediment flux obtained from the pore water concentration of CH_4 $F_{sed,Hal}$ = 2.8 mmol m^{-2} d^{-1}. Hence, in contrast to the conclusion of Donis et al. [1], sediment fluxes provide sufficient CH_4 to compensate losses due to emissions from the lake surface.

Balancing atmospheric emissions (1800 to1900 mol d^{-1}) with the total source due to sediment fluxes (1990 mol d^{-1}) suggests an overall net sink of CH_4 in the surface mixed layer of Lake Hallwil of 140 ± 50 mol d^{-1} which is consistent with the typical oxidation rate of CH_4 of ~150 mol d^{-1} cited in Donis et al. [1]. The data clearly does not support the claim by Donis et al. [1] that the balance between emissions and sediment fluxes in Lake Hallwil implies a missing source of almost 4800 mol d^{-1}. The conclusion by Donis et al. [1] that the overall mass balance requires a CH_4 production rate of 4600 mol d^{-1} in the surface mixed layer is inconsistent with their data.

Section S4: Sensitivity of the estimated sediment fluxes $F_{Sed,S}$ to the choice of the empirical relation for the wind dependence of the gas-transfer velocity

The estimates of diffusive emissions from the lake surface depend on the gas transfer velocity (see equ. S3). The gas transfer velocity measures the exchange rate across the diffusive boundary layer at the lake surface. The thickness of this boundary layer and the transport within this layer depends on viscosity, the molecular diffusivity of the gas and on the turbulence in the surface waters (see e.g. [22]). Viscosity and molecular diffusivity depend on water temperature and are considered via the Schmidt number of the gas at the temperature of the surface water (see equ. S3). The effects of turbulence in the water column on the gas-transfer velocity are typically parameterized via wind speed. Several empirical relations are available, e.g. [4,19–21,23]. The results of our analysis are based on the model of Cole and Caraco (main text Figure 1a and Figure S9a) and are compared here to results obtained from the models of Guernin et al. (Figure S9c) and MacIntyre et al. (Figure S9b,d). MacIntyre et al. [23] have distinguished between gas exchange velocities under conditions of positive and negative surface buoyancy flux. During time periods of negative surface buoyancy flux, i.e. the lake loses heat to the atmosphere, convection is induced generating near surface transport and turbulence in addition to the wind induced turbulence. Hence, gas transfer velocities during time periods of surface cooling are larger than during time periods of surface warming. MacIntyre et al. [23] provide three parameterizations of the gas transfer velocity as function wind speed: for periods of (a) positive surface buoyancy flux, (b) negative surface buoyancy flux, and (c) positive and negative surface buoyancy fluxes combined. Most of our data on basin wide distributions of CH_4 were collected in spring and early summer when the lakes are warming and condition (a) applies. Results using the model of [23] for positive surface buoyancy flux are shown in Figure S9b. However, in late summer and fall night time convection may occur and condition (c), positive (during day time) and negative (during night time) surface buoyancy fluxes may be important. Results using the model of [23] assuming positive surface buoyancy fluxes between March and July (parametrization a) and positive and negative surface buoyancy fluxes in the rest of the year (parametrization c) are shown in Figure S9d as MacIntyre et al. [23] (mixed model).

All parameterizations investigated show the same result: $F_{sed,S}$ required to compensate emissions at 20°C is on average slightly smaller than the sediment flux $F_{sed,Hal}$ determined from pore-water measurements in Lake Hallwil using the same data and the same approach as Donis et al. [1]. The claim that emissions exceed sediment fluxes by a factor of 26 [1] is not supported by our data and estimate.

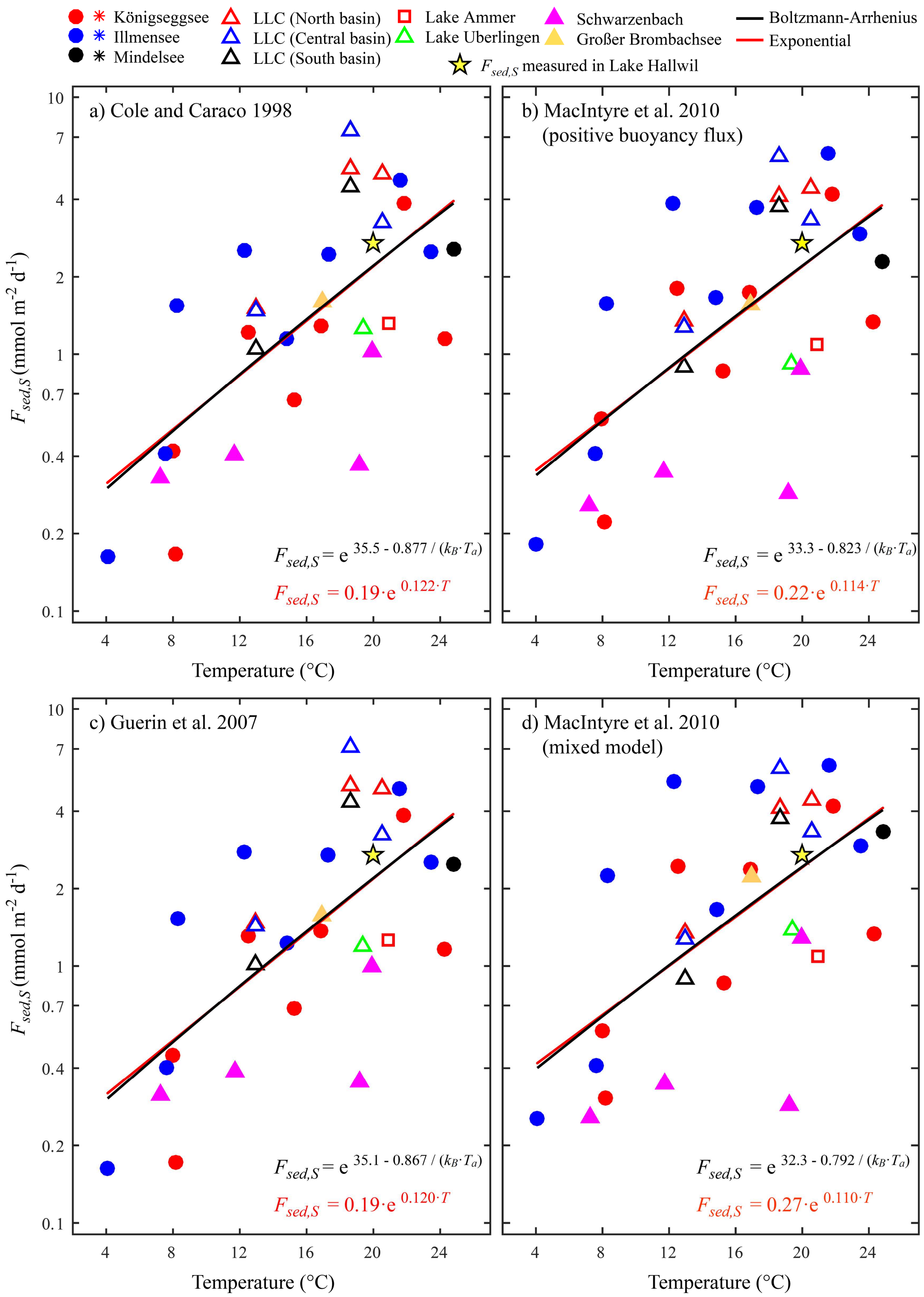

Figure S9: $F_{sed,S}$ required to compensate diffusive CH_4 emissions from different lakes and in different seasons. Diffusive CH_4 emissions are calculated based on the basin average surface CH_4 concentration and the gas exchange model (equ. S3) using different parameterizations of the gas transfer velocity as function for wind speed: (a) Cole and Caraco 1998 [4]; (b) MacIntyre et al 2010 (positive buoyancy flux) [23]; (c) Guerin et al. 2007 [19]; (d) MacIntyre et al. 2010 (mixed model) [23].

Section S5. References cited in the supplement

1. Donis, D. *et al.* Full-scale evaluation of methane production under oxic conditions in a mesotrophic lake. *Nat. Commun.* **8,** 1–11 (2017).
2. Hofmann, H., Federwisch, L. & Peeters, F. Wave-induced release of methane: Littoral zones as source of methane in lakes. *Limnology and Oceanography* **55,** 1990–2000 (2010).
3. Encinas Fernández, J., Peeters, F. & Hofmann, H. On the methane paradox: Transport from shallow water zones rather than in situ methanogenesis is the mayor source of CH4 in the open surface water of lakes. *J. Geophys. Res. Biogeosciences* **121,** 2717–2726 (2016).
4. Cole, J. J. & Caraco, N. F. Atmospheric exchange of carbon dioxide in a low-wind oligotrophic lake measured by the addition of SF6. *Limnol. Oceanogr.* **43,** 647–656 (1998).
5. Schilder, J. *et al.* Spatial heterogeneity and lake morphology affect diffusive greenhouse gas emission estimates of lakes. *Geophys. Res. Lett.* **40,** 5752–5756 (2013).
6. Liss, P. S. & Merlivat, L. in *The Role of Air−Sea Exchange in Geochemical Cycling* (ed. Reidel, D.) 113–127 (Publishing Company, Dordrecht, The Netherlands, 1986).
7. Wanninkhov, K. Relationship between wind speed and gas exchange. *J. Geophys. Res. Ocean.* **97,** 7373–7382 (1992).
8. Bogard, M. J. *et al.* Oxic water column methanogenesis as a major component of aquatic CH4 fluxes. *Nat. Commun.* **5,** 5350 (2014).
9. DelSontro, T., Del Giorgio, P. A. & Prairie, Y. T. No Longer a Paradox: The Interaction Between Physical Transport and Biological Processes Explains the Spatial Distribution of Surface Water Methane Within and Across Lakes. *Ecosystems* 1–15 (2017). doi:10.1007/s10021-017-0205-1
10. Paranaíba, J. R. *et al.* Spatially Resolved Measurements of CO2and CH4Concentration and Gas-Exchange Velocity Highly Influence Carbon-Emission Estimates of Reservoirs. *Environ. Sci. Technol.* **52,** 607–615 (2018).
11. Wiesenburg, D. A. & Guinasso, N. L. Equilibrium Solubilities of Methane, Carbon Monoxide, and Hydrogen in Water and Sea Water. *J. Chem. Eng. Data* **24,** 356–360 (1979).
12. Lawrence, G. A., Ashley, K. I., Yonemitsu, N. & Ellis, J. R. Natural dispersion in a small lake. *Limnol. Oceanogr.* **40,** 1519–1526 (1995).
13. Okubo, A. Oceanic diffusion diagrams. *Deep. Res.* **18,** 789–802 (1971).
14. Peeters, F. & Hofmann, H. Length-scale dependence of horizontal dispersion in the surface water of lakes. *Limnol. Oceanogr.* **60,** 1917–1934 (2015).
15. Ribi, B., Bührer, H. & Ambühl, H. Hypsographische Daten von Seen Zusammenstellung der hypsographischen Daten. *http://www.hbuehrer.ch/pdf/Seetopo.pdf* 1–42 (2005).

16. Yvon-Durocher, G. *et al.* Methane fluxes show consistent temperature dependence across microbial to ecosystem scales. *Nature* **507,** 488–491 (2014).

17. Rasilo, T., Prairie, Y. T. & del Giorgio, P. A. Large-scale patterns in summer diffusive CH_4 fluxes across boreal lakes, and contribution to diffusive C emissions. *Glob. Chang. Biol.* **21,** 1124–1139 (2015).

18. MacIntyre, S. *et al.* Buoyancy flux, turbulence, and the gas transfer coefficient in a stratified lake. *Geophys. Res. Lett.* **37,** 2–6 (2010).

19. Guérin, F. *et al.* Gas transfer velocities of CO_2 and CH_4 in a tropical reservoir and its river downstream. *J. Mar. Syst.* **66,** 161–172 (2007).

20. Schwarzenbach, R. P., Gschwend, P. M. & Imboden, D. M. *Environmental organic chemistry*. (A John Wiley & Sons, 2003).

21. Crusius, J. & Wanninkhof, R. Gas transfer velocities measured at low wind speed over a lake. *Limnol. Oceanogr.* **48,** 1010–1017 (2003).

22. Lorke, A. & Peeters, F. Toward a Unified Scaling Relation for Interfacial Fluxes. *Journal of Physical Oceanography* **36,** 955–961 (2006).

23. MacIntyre, S. *et al.* Buoyancy flux, turbulence, and the gas transfer coefficient in a stratified lake. *Geophys. Res. Lett.* **37,** 93106 (2010).
